# Crosstalk in oxygen homeostasis networks: SKN-1/NRF inhibits the HIF-1 hypoxia-inducible factor in *Caenorhabditis elegans*

**DOI:** 10.1371/journal.pone.0249103

**Published:** 2021-07-09

**Authors:** Dingxia Feng, Zhiwei Zhai, Zhiyong Shao, Yi Zhang, Jo Anne Powell-Coffman

**Affiliations:** Department of Genetics, Development and Cell Biology, Iowa State University, Ames, Iowa, United States of America; All India Institute of Medical Sciences, INDIA

## Abstract

During development, homeostasis, and disease, organisms must balance responses that allow adaptation to low oxygen (hypoxia) with those that protect cells from oxidative stress. The evolutionarily conserved hypoxia-inducible factors are central to these processes, as they orchestrate transcriptional responses to oxygen deprivation. Here, we employ genetic strategies in *C*. *elegans* to identify stress-responsive genes and pathways that modulate the HIF-1 hypoxia-inducible factor and facilitate oxygen homeostasis. Through a genome-wide RNAi screen, we show that RNAi-mediated mitochondrial or proteasomal dysfunction increases the expression of hypoxia-responsive reporter *Pnhr-57*::*GFP* in *C*. *elegans*. Interestingly, only a subset of these effects requires *hif-1*. Of particular importance, we found that *skn-1* RNAi increases the expression of hypoxia-responsive reporter *Pnhr-57*::*GFP* and elevates HIF-1 protein levels. The SKN-1/NRF transcription factor has been shown to promote oxidative stress resistance. We present evidence that the crosstalk between HIF-1 and SKN-1 is mediated by EGL-9, the prolyl hydroxylase that targets HIF-1 for oxygen-dependent degradation. Treatment that induces SKN-1, such as heat or *gsk-3* RNAi, increases expression of a *Pegl-9*::*GFP* reporter, and this effect requires *skn-1* function and a putative SKN-1 binding site in *egl-9* regulatory sequences. Collectively, these data support a model in which SKN-1 promotes *egl-9* transcription, thereby inhibiting HIF-1. We propose that this interaction enables animals to adapt quickly to changes in cellular oxygenation and to better survive accompanying oxidative stress.

## Introduction

Oxygen homeostasis has profound effects on health and fitness. Oxygen serves as the terminal electron acceptor in the oxidative phosphorylation processes that generate energy for life. When oxygen levels are low (hypoxia), cells and tissues must adapt quickly by increasing oxygen delivery, adjusting the levels of key metabolic enzymes, and limiting the accumulation of misfolded proteins. While oxygen is essential, it is also highly reactive. The reactive oxygen species (ROS) generated by cellular metabolism and signaling processes can damage macromolecules, and excess ROS are thought to contribute to cellular aging and deterioration [1–3]. One of the central challenges of aerobic life is to coordinate the biological networks that control disparate aspects of oxygen homeostasis.

This balance between surviving hypoxic stress and mitigating the potential damage caused by reactive oxygen species is especially important in cardiovascular development and disease. When ischemia blocks circulation to a mammalian tissue, oxygen levels drop, and cells induce hypoxia-inducible transcription factors (HIFs). Upon reperfusion and reoxygenation of the tissue, mammalian cells respond by rapidly degrading HIF and inducing the NRF2 transcription factor [4, 5]. However, intermittent hypoxia has been shown to induce both HIF-1α and NRF2 [6, 7]. NRF2 activates phase II detoxification genes to mitigate the effects of oxidative insults [8, 9]. Although mammalian HIF and NRF2 share some common target genes such as aldehyde dehydrogenase 1A1 or heme oxygenase-1 HO-1, the genes induced by re-oxygenation are largely distinct from those that respond to oxygen deprivation [5, 10, 11]. Crosstalk between these two pathways is complex and context specific in mammals [12], as these important transcription factors facilitate the rapid changes in gene expression needed to limit reperfusion injury and regulate oxygen-dependent developmental processes.

*C*. *elegans* has been proven to be an excellent model system for studying the regulatory networks that govern oxygen homeostasis. The *C*. *elegans* genome encodes a single hypoxia-inducible factor alpha subunit (HIF-1), and the *hif-1* gene has been shown to have important roles in stress response and in aging [13–17]. HIF protein levels and HIF activity are tightly regulated. When oxygen is abundant, the HIF alpha subunit is hydroxylated by the PHD/EGL-9 enzymes. Once modified, HIFα protein interacts with the Von Hippel-Lindau tumor suppressor (VHL) and is targeted for ubiquitination and proteasomal degradation [18–23]. Thus, in hypoxic conditions, HIF-1 protein is stable, and the transcription factor complex can activate the expression of a battery of genes that enable adaptation to low oxygen. This pathway for oxygen-dependent degradation of HIF protein is evolutionarily conserved. The *C*. *elegans hif-1*, *aha-1*, *egl-9*, and *vhl-1* genes are orthologous to mammalian *HIFα*, *HIFβ*, *PHD*, and *VHL*, respectively [24–26]. The targets of *C*. *elegans* HIF-1 include *egl-9* and *rhy-1*, genes that inhibit HIF-1 expression and activity [27–30]. In wild-type animals, these negative feedback loops keep HIF-1 activity in check and limit the potentially adverse effects of HIF-1 over-activation.

The *C*. *elegans skn-1* gene is homologous to mammalian *NRF1/2/3* [31]. SKN-1 regulates the expression of a battery of genes with cytoprotective functions, including phase II detoxification genes [32, 33]. SKN-1 is activated by a range of stresses or toxicants that cause oxidative stress, and SKN-1 promotes resistance to these insults [32, 34–37].

Here, we investigate the cellular processes and transcriptional networks that regulate HIF-1 function. We describe an unbiased RNAi screen to identify genes that inhibit *C*. *elegans* HIF-1. This approach builds upon and extends mutational screens that identified negative regulators of HIF-1 [17, 29]. We discover that SKN-1/NRF represses HIF-1 protein levels. Hence, SKN-1-mediated repression of HIF-1 may provide a mechanism by which cells can rapidly respond to specific environmental stresses and optimize gene expression to achieve oxygen homeostasis. We investigate the hypothesis that this cross talk is mediated by EGL-9, the oxygen-sensing prolyl hydroxylase that modulates HIF-1 stability and activity.

## Results

**Fig 1:**
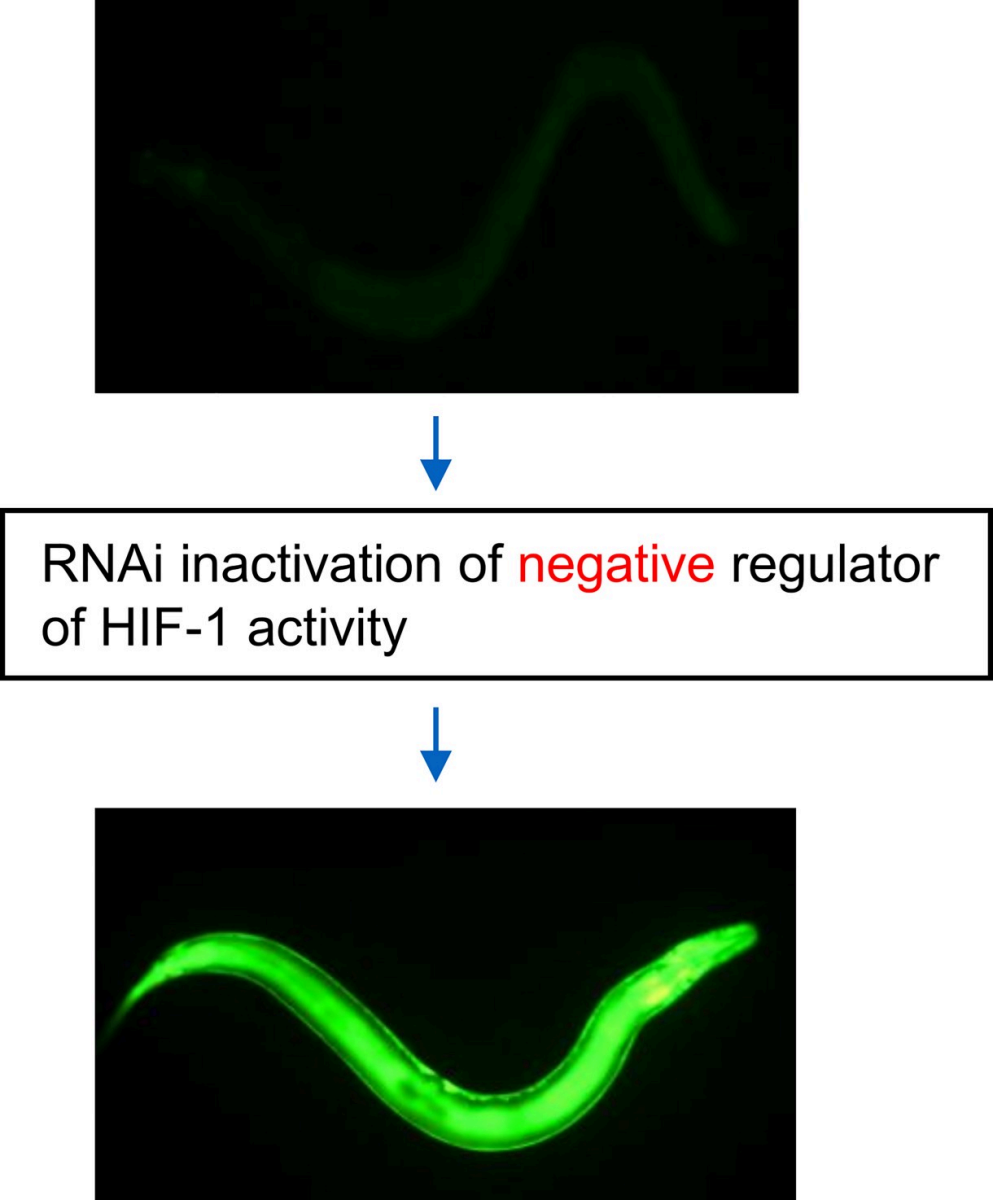
Genome-wide RNAi screen to identify negative regulators of HIF-1-mediated gene expression. Illustration of screen design. *C*. *elegans* expressing the *Pnhr-57*::GFP reporter were fed bacteria expressing gene-specific RNAi. Prior studies had shown that this reporter is induced by hypoxia and is positively regulated by the HIF-1 transcription factor. In controls (photo at the top), animals exhibited a low level of fluorescence, while RNAi treatments that increased expression of the reporter resulted in high levels of fluorescence.

To identify genes and cellular processes that attenuated HIF-1-mediated gene expression, we conducted a genome-wide RNAi screen. This experimental strategy relied on the *Pnhr-57*::*GFP* reporter gene, which had been shown to be responsive to HIF-1 and hypoxia [28, 29]. Through chromatin immunoprecipitation experiments, we confirmed that *nhr-57* was a direct target of HIF-1 (S1 Fig). We screened a bacterial RNAi library representing ~80% of *C*. *elegans* genes [38], and we identified 179 genes for which RNAi increased *Pnhr-57*::*GFP* expression, as assayed by inspection under a fluorescent stereomicroscope (screen design illustrated in Fig 1). These genes and their related functions are listed in S1 Table. Among these genes, the most enriched biological terms are proteasome (23 genes, 13%, *p*-value = 1.38E-37) and mitochondrion (39 gene, 22%, *p*-value = 4.49E-37). Table 1 lists the top 10 most enriched biological terms associated with this gene list.

**Table 1:** Top 10 enriched biological terms for the 179 genes that increased *Pnhr-57*::GFP expression when knocked-down by RNAi.

Biological term	Count	%	*p*-value
Proteasome	23	13	1.38E-37
Mitochondrion	39	22	4.49E-37
Transit peptide	23	13	9.57E-23
Mitochondrion inner membrane	17	10	2.58E-18
Ribosomal protein	21	12	3.07E-18
Ribonucleoprotein	21	12	1.25E-16
Transport	31	18	8.99E-13
Hydrogen ion transport	9	5	3.63E-11
Electron transport	10	6	7.64E-11
Threonine protease	7	4	3.39E-10

Recognizing that most eukaryotic genes are coordinately regulated by multiple transcription factors, we did a secondary screen to identify those RNAi treatments that had a clear *hif-1*-dependent effect on *Pnhr-57*::*GFP* expression. To do this, we compared the *Pnhr-57*::*GFP* induction of these 179 RNAi treatments in wild-type animals and *hif-1*-deficient animals. Most of the 179 RNAi treatments increased the expression of *Pnhr-57*::*GFP* independent of *hif-1*. However, the *Pnhr-57*::*GFP* induction by 13 RNAi treatments showed a strong *hif-1*-dependent effect (S2 Table). Among these 13 genes, as expected, RNAi for *egl-9*, *rhy-1*, and *vhl-1*, previously characterized negative regulators of *C*. *elegans* HIF-1 [26, 29], increased *Pnhr-57*::*GFP* expression in wild-type animals, but not in *hif-1* mutants. These results validated the efficacy of our screen approach, and gave us the confidence to continue investigating the potentially new negative regulators of HIF-1 among these 13 genes.

### *skn-1* attenuates HIF-1 protein levels and HIF-1 function

**Fig 2:**
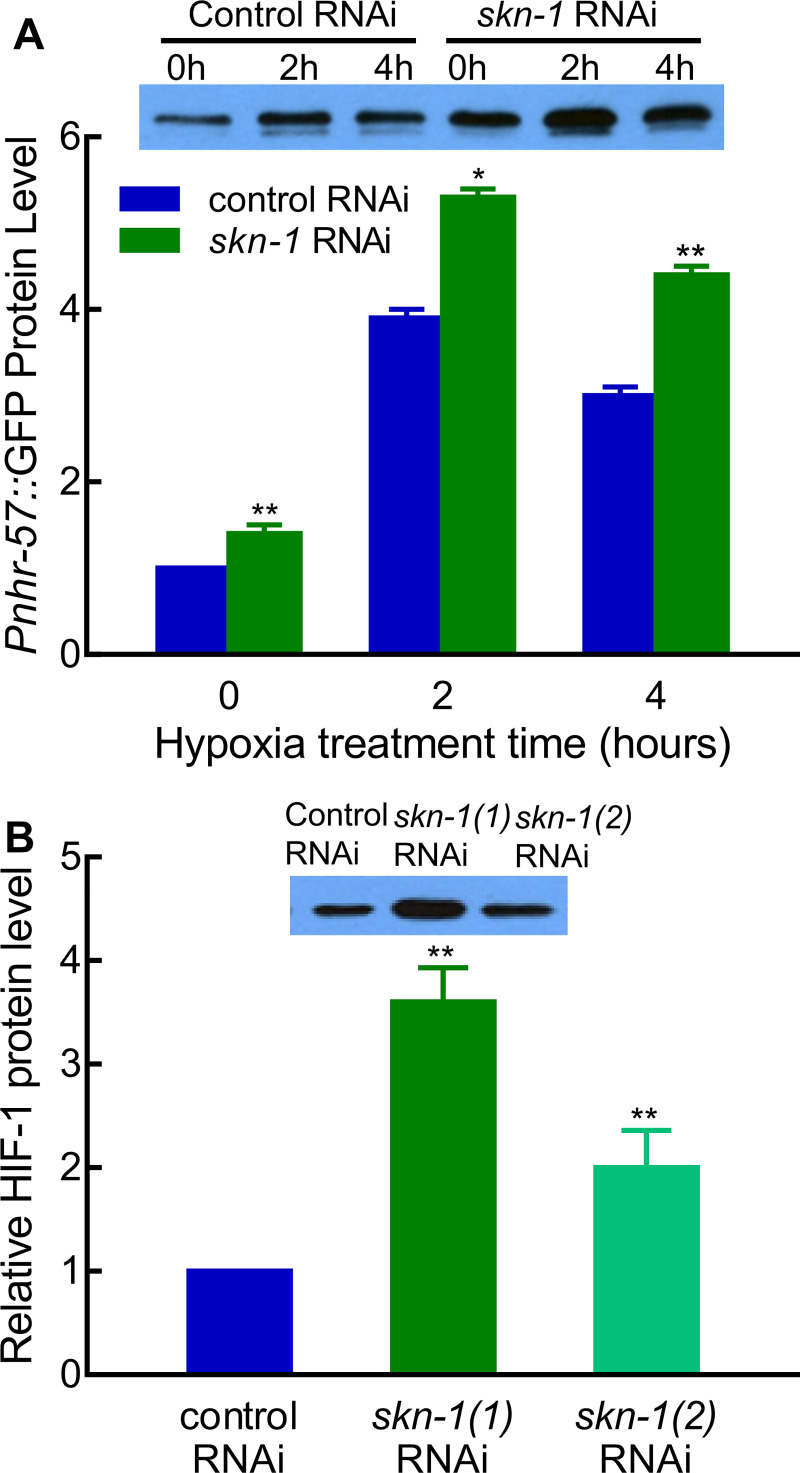
Identification of SKN-1 as a regulator of HIF-1. (A) *skn-1* RNAi increased expression of the *Pnhr-57*::*GFP* reporter. Reporter gene expression was quantitated in L4-stage animals in normal culture conditions or after 2 or 4 hours of hypoxia treatment (0.5% oxygen). The protein levels were calculated from three independent experiments and normalized to 0 hour hypoxia control RNAi. A representative western blot is shown. In each lane, lysates from 80 L4-satge worms were loaded. Asterisks indicate significant differences between control RNAi and *skn-1* RNAi at any given time point. *: *p* < 0.05; **: *p* < 0.01. (B) *skn-1* RNAi increased HIF-1 protein levels. These animals expressed an epitope-tagged HIF-1 protein [40]. The protein levels were calculated from three independent experiments and normalized to control RNAi. Two different RNAi clones were assayed, and they are designated as *skn-1(1)* and *skn-1(2)* here. A representative western blot is shown. In each lane, lysates from 100 L4-satge worms were loaded. Asterisks indicate significant differences between control RNAi and *skn-1* RNAi at any given time point. **: *p* < 0.01.

We were especially intrigued by the finding that *skn-1* RNAi increased the expression of HIF-1-responsive reporter. The Transcription factor SKN-1 has been shown to have critical roles in enabling *C*. *elegans* to respond to oxidative stress [32–34, 39]. Our finding suggested a potential crosstalk between hypoxia response and oxidative stress response. To quantify the effect of *skn-1* RNAi on this HIF-1-responsive reporter, we examined *Pnhr-57*::GFP levels using protein blots. In the normal room air culture conditions, *Pnhr-57*::GFP was 40% higher in *skn-1* RNAi compared to control RNAi (Fig 2A) (**p < 0.01, from three independent experiments). To gain insight to the effects of this interaction in hypoxic conditions, we moved the animals to 0.5% oxygen. After 4 hours of hypoxia treatment, *Pnhr-57*::GFP was 50% higher in *skn-1* RNAi compared to control RNAi (Fig 2A) (**p < 0.01, from three independent experiments). Thus, *skn-1* RNAi increased *Pnhr-57*::GFP levels under normoxic and hypoxic conditions.

We next asked whether *skn-1* RNAi increased HIF-1 protein levels. We tested two *skn-1* RNAi constructs, and each resulted in an increase of HIF-1 protein levels by 2 to 3-fold as shown in Fig 2B (**p < 0.01, from three independent experiments). In sum, these results showed that *skn-1* RNAi increased HIF-1 protein level and HIF-1 reporter expression.

### Differential requirements for *skn-1* and *hif-1*

The finding that SKN-1 repressed HIF-1 protein levels suggested that there might be conditions in which it would be beneficial for the animal to express one of these two stress-responsive transcription factors, but not the other. To address this, we examined the relative requirements for *skn-1* and *hif-1* more closely.

In previous studies, we and others had shown that *hif-1* was required for survival in moderate hypoxia [25, 41]. As validated in the experiments described in Table 2, loss of *hif-1* impaired animal development and survival in 0.5% oxygen: after 24 hours of hypoxia treatment, only 75.8% of *hif-1*-deficient eggs hatched, and only 25.6% developed to adulthood within 72 hours. In contrast, *skn-1* RNAi had no effect on *C*. *elegans* development and survival in hypoxic conditions: after 24 hours of hypoxia treatment, 99.4% of *skn-1* RNAi treated eggs hatched and completed normal development to adulthood within 72 hours.

**Table 2:** Relative requirements for *skn-1* and *hif-1*: Survival in 0.5% oxygen.

Genotype	% hatched ± SEM	% survive to adult ± SEM	n ^a^
N2 (wild type)	99.1 ± 0.25	99.1± 0.3	672
*hif-1(ia04)*	75.8 ± 2.5	25.6 ± 0.9	616
N2;control(RNAi)	99.6 ± 0.19	99.6 ± 0.2	558
N2;*skn-1(RNAi)*	99.4 ± 0.42	99.4 ± 0.4	640

^a^ n is the total number of animals assayed in three independent experiments.

Prior studies also suggested that there were differential requirements for HIF-1 and SKN-1 in oxidative stress conditions. Mutants carrying loss-of-function mutations in *skn-1* have been shown to decrease the ability of *C*. *elegans* to survive exposure to agents that cause oxidative stress [34, 42–44]. In contrast, *C*. *elegans* carrying loss-of-function mutations in *hif-1* have been reported to be relatively resistant to peroxide [40]. We compared these phenotypes directly, and the data are provided in Table 3. These experiments confirmed that, while *skn-1*-deficient animals were sensitive to t-butyl peroxide, mutants lacking *hif-1* were remarkably resistant to this oxidative stress: while none of the *skn-1-*deficient mutants survived 6 hours of t-butyl peroxide treatment, 97.5% of *hif-1*-deficient mutants survived 10 hours of t-butyl peroxide treatment.

**Table 3:** Relative requirements for *skn-1* and *hif-1*: Survival on t-butyl-peroxide.

Exposure time	Genotype	Mean Survival ± SEM	N ^a^
6 hours	N2 (wild type)	95.2 ± 2.6	106
	*skn-1(zu67)*	0.0 ± 0	117
	*hif-1(ia04)*	100.0 ± 0	120
8 hours	N2 (wild type)	29.08 ± 7.7	106
	*skn-1(zu67)*	0.0 ± 0	117
	*hif-1(ia04)*	99.17 ± 0.8	120
10 hours	N2 (wild type)	2.78 ± 2.8	106
	*skn-1(zu67)*	0.0 ± 0	117
	*hif-1(ia04)*	97.50 ± 2.5	120

^a^ n is the total number of animals assayed in three independent experiments.

### SKN-1/NRF promotes *egl-9* expression

**Fig 3:**
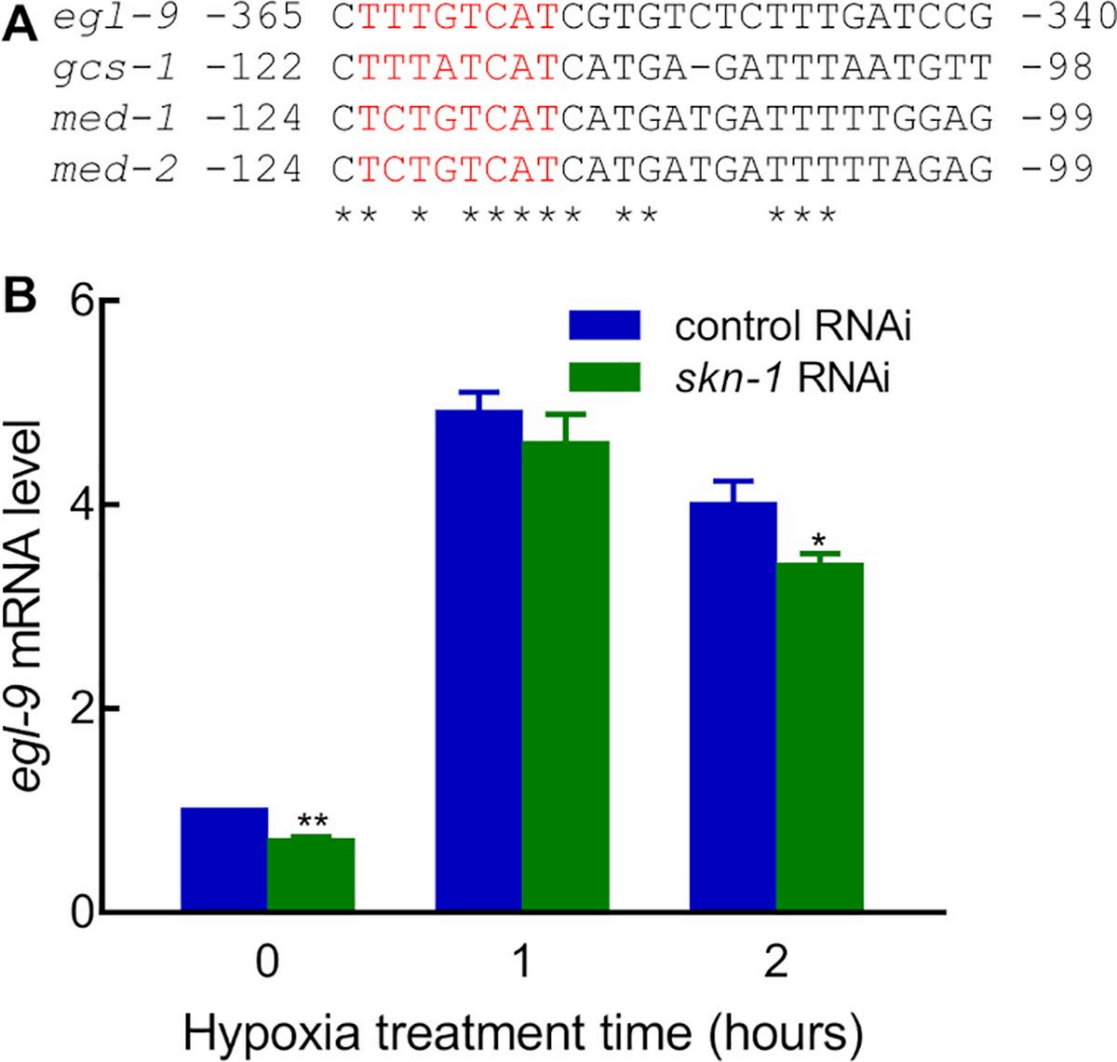
Identification of *egl-9* as a potential transcriptional target of SKN-1. (A) Sequence from the *egl-9* promoter was aligned with established SKN-1 binding sites in *gcs-1*, *med-1*, and *med-2*. Asterisks identify sequence identities shared by all four promoter regions in this interval, and predicted SKN-1 binding sites are in red. (B) *skn-1* RNAi decreased *egl-9* mRNA levels. *egl-9* mRNA levels were quantitated from three independent real-time quantitative RT-PCR experiments. The values at each time point were normalized to the 0 hour hypoxia control RNAi. Asterisks indicate significant differences between control RNAi and *skn-1* RNAi at any given time point. *: *p* < 0.05; **: *p* < 0.01.

We next sought to discover the mechanism by which SKN-1 inhibits HIF-1 protein levels. In silico analyses identified a potential SKN-1 binding site in the *egl-9* promoter region (Fig 3A). EGL-9 is a central inhibitor of HIF-1 protein levels [26, 27] and of HIF-1 transcriptional activity [29, 30]. This suggested a model in which the SKN-1 DNA-binding complex bound directly to the *egl-9* regulatory sequences to promote *egl-9* expression, which, in turn, would ultimately decrease HIF-1 protein levels. To test this, we employed real-time quantitative RT-PCR to compare *egl-9* mRNA levels in worms fed with *skn-1* RNAi versus control RNAi. To produce reliable and reproducible results, *egl-9* mRNA levels were quantitated in three independent real-time quantitative RT-PCR experiments in L4-stage animals in room air or hypoxic conditions (0.5% oxygen). Each sample was performed with three technical replicates, and they produced similar C_t_ values. There are seven isoforms of *egl-9* mRNA transcripts (https://wormbase.org/species/c_elegans/gene/WBGene00001178#0-9f-10). The real-time quantitative PCR primer set used in this study can detect six *egl-9* mRNA isoforms. In room air, *skn-1* RNAi decreased *egl-9* mRNA levels by 30% compared to control RNAi (**p < 0.01, from three independent experiments) (Fig 3B). HIF-1 has been shown to activate *egl-9* mRNA expression under hypoxia, creating a negative feedback loop [27, 28]. In accordance with this, the inhibition effects of *skn-1* RNAi on *egl-9* mRNA levels were minimized by placing the animals in hypoxic conditions (Fig 3B).

**Fig 4:**
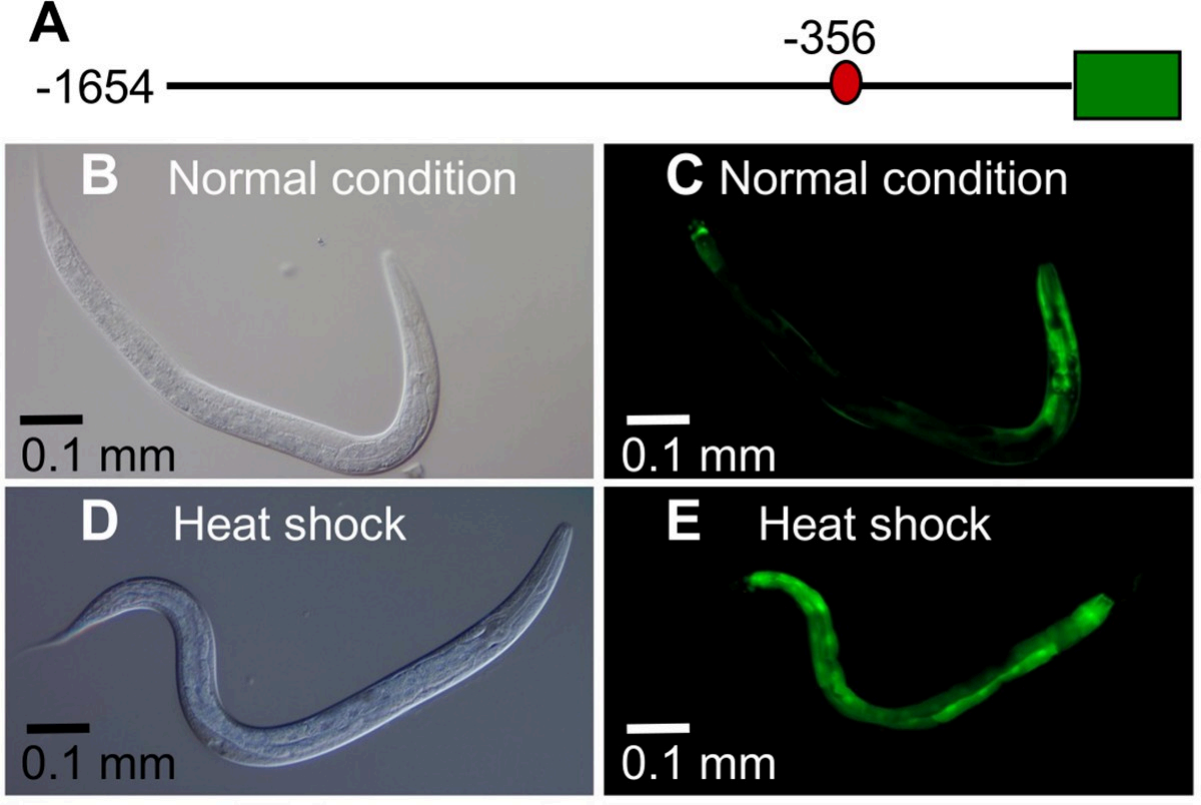
Heat shock alters *Pegl-9*::*GFP* expression. (A) The *Pegl-9*::*GFP* construct includes 1.6 kb of sequence 5’ to the *egl-9* translational start. GFP coding sequence is diagramed as a green box. The red oval indicates the position of the putative SKN-1 binding site. (B-E) *Pegl-9*::*GFP* expression in L4-stage animals under normal culture conditions and heat shock. Animals are shown as DIC images (B and D) and corresponding images of GFP fluorescence (C and E). In all images, the head is to the right. (B and C) Under normal conditions, *Pegl-9*::*GFP* was expressed in the body muscle, vulva, pharynx, anterior intestine, rectal cells and additional cells in the tail. (D and E) After heat shock treatment (29°C for 20 hours), *Pegl-9*::*GFP* was strongly induced in the intestine. The*Pegl-9*::*GFP* expression patterns in the L1, L2, L3 and adults were similar to that in the L4 worms, under both normal and heat shock conditions (S2 Fig).

To test the hypothesis that conditions that activate SKN-1 can promote *egl-9* promoter activity, we generated a reporter construct in which 1.6 kb of *egl-9* regulatory sequence directed the expression of GFP (Fig 4A). To distinguish the effects of SKN-1 on *egl-9* expression from those of HIF-1, we conducted these experiments in a *hif-1* mutant background. In agreement with prior studies [45], *Pegl-9*::GFP was visible in several tissues, including the body muscle, vulva, pharynx, anterior intestine, rectal cells and additional cells in the tail in standard culture conditions (20°C) (Fig 4B and 4C). When the animals were treated with heat shock conditions that had been shown to activate SKN-1 (29°C for 20 hours) [34], we observed dramatic induction of *Pegl-9*::GFP in the intestine. (Fig 4D and 4E).

**Fig 5:**
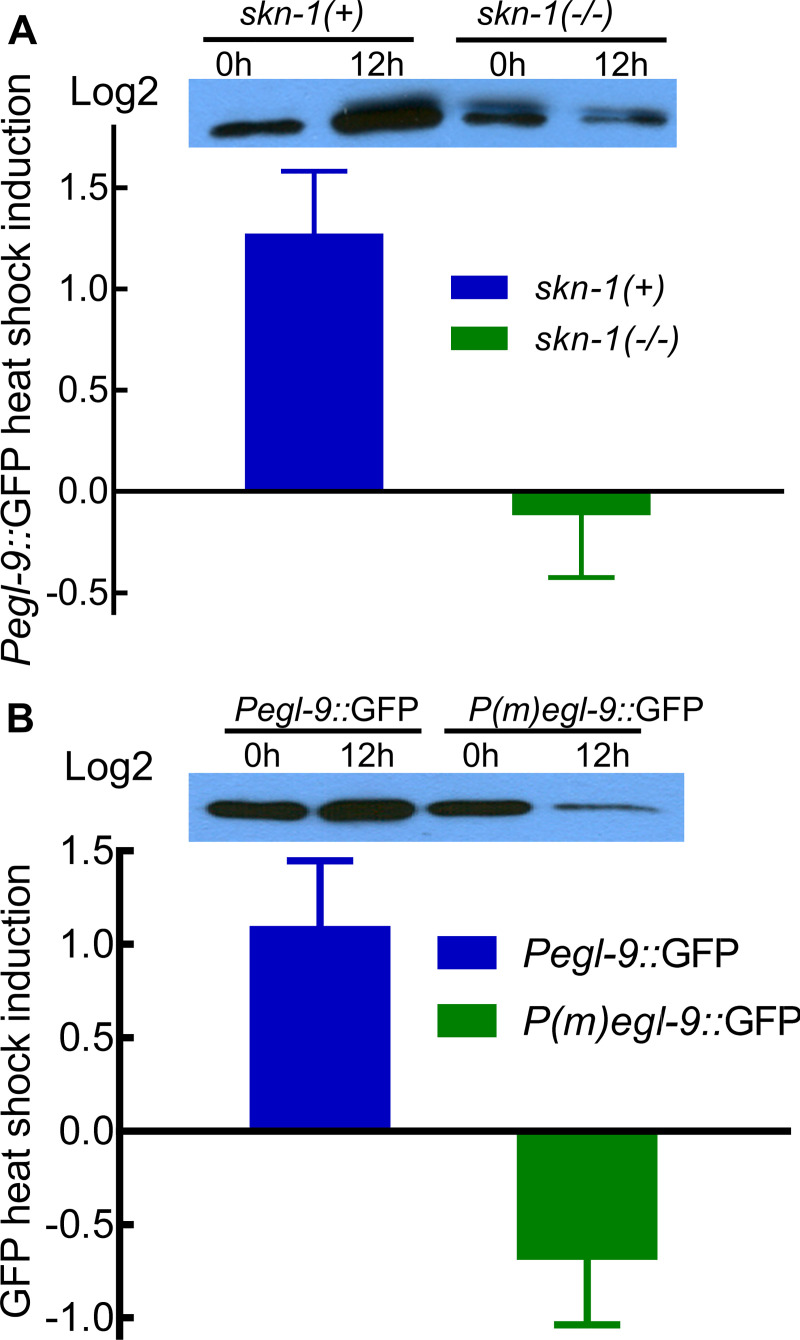
SKN-1 acts through the putative SKN-1 binding site in the *egl-9* promoter to activate *egl-9* expression. (A) Heat shock induced *Pegl-9*::*GFP* in animals carrying the wild-type s*kn-1* allele, but did not induce the reporter in animals carrying the *skn-1(zu67)* loss-of-function mutation. The vertical axis shows the log2 fold changes of GFP caused by heat shock for each strain with standard errors, as determined by four biological replicates. A representative western blot is shown. For each sample, 20 L4- stage worms were boiled and lysates corresponding to 10 worms were loaded to each lane. (B) Heat shock increased the expression of *Pegl-9*::*GFP*, but did not increase the expression of the reporter in which the putative SKN-1 binding site was mutated (*P(m)egl-9*::*GFP*). The vertical axis shows the log2 fold changes of GFP caused by heat shock for each strain with standard errors, as determined by five biological replicates. A representative western blot is shown. For each sample, 20 L4- stage worms were boiled and lysates corresponding to 10 worms were loaded to each lane.

We next asked whether heat shock induction of *Pegl-9*::*GFP* required *skn-1* function. We found that heat shock increased *Pegl-9*::GFP by 2.5-fold in animals carrying the wild-type *skn-1* allele. However, the heat shock induction of *Pegl-9*::*GFP* was abolished in *skn-1(zu67)* loss-of-function mutants (Fig 5A). Analyses of another independent *Pegl-9*::*GFP* transgenic line yielded similar results (S3 Fig).

To test the hypothesis that the putative SKN-1 binding site in the *egl-9* promoter was required for heat shock induction of *Pegl-9*::*GFP*, we generated the *P(m)egl-9*::*GFP* construct, which contained mutations in the putative SKN-1 binding site (in red type in Fig 3A). Heat shock increased *Pegl-9*::GFP by 2.1-fold. However, heat shock failed to induce the expression of *P(m)egl-9*::*GFP* (Fig 5B). Experiments with a second *P(m)egl-9*::*GFP* transgenic line gave similar results (S4 Fig). Collectively, these data demonstrated that heat shock induction of *Pegl-9*::*GFP* required *skn-1* function and the putative SKN-1 binding site in the *egl-9* promoter.

**Fig 6:**
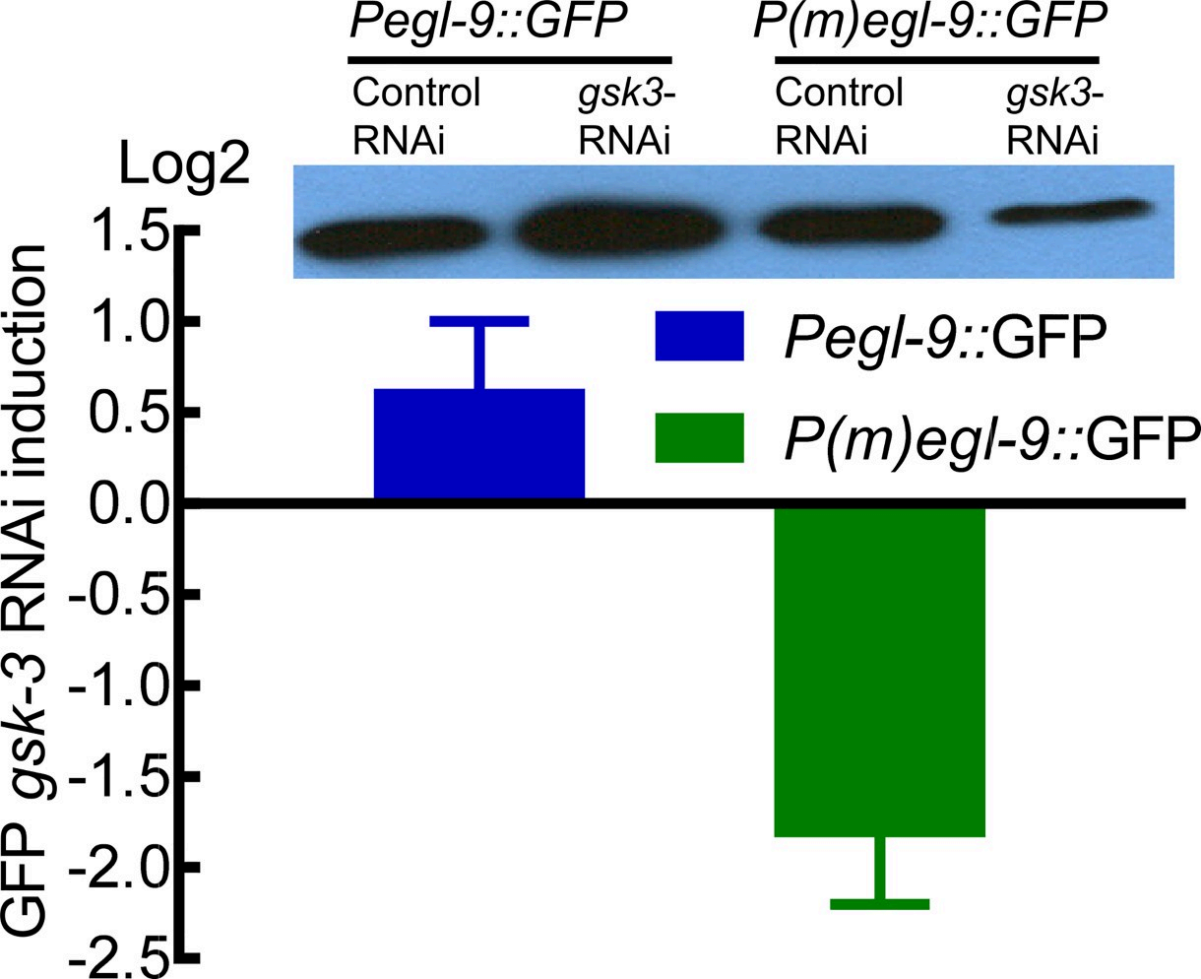
*gsk-3* RNAi induction of *Pegl-9*::GFP. *gsk-3* RNAi increased expression of *Pegl-9*::GFP, relative to control RNAi (the L4440 empty vector). This effect was dependent upon the putative SKN-1 binding site in the reporter (mutated in *P(m)egl-9*::*GFP*). The figure shows the log2 fold change of GFP from four biological replicates, with standard errors. A representative western blot is shown. For each sample, 20 L4-stage worms were boiled and lysates corresponding to 10 worms were loaded to each lane.

**Fig 7:**
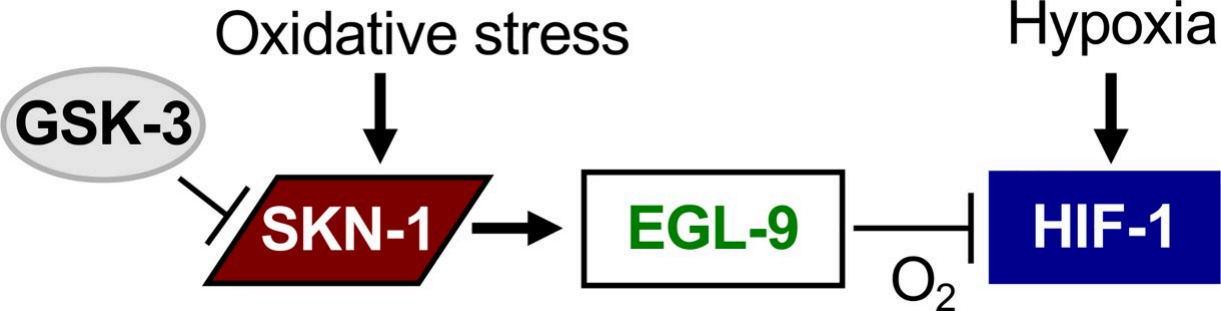
SKN-1 regulates *egl-9* expression to attenuate HIF-1. Model illustrating interactions between key regulators of oxygen homeostasis in *C*. *elegans*. SKN-1 promotes *egl-9* transcription, and EGL-9 controls oxygen-dependent degradation of HIF-1 protein.

We employed *gsk-3* RNAi as an independent means of activating SKN-1. GSK-3 (glycogen synthase kinase-3) is a negative regulator of SKN-1. Under normal conditions, SKN-1 is present at low levels in intestinal nuclei. Prior studies had demonstrated that *gsk-3* RNAi caused constitutive expression of SKN-1 in intestinal nuclei in the absence of oxidative stress [39, 46]. As shown in Fig 6, *gsk-3* RNAi increased the expression of *Pegl-9*::GFP by 1.5-fold. Notably, *gsk-3* RNAi failed to induce the expression of *P(m)egl-9*::GFP, in which the SKN-1 binding site is disrupted. Collectively, these data support a model in which SKN-1 promotes the transcription of *egl-9*, thereby repressing HIF-1 (Fig 7).

## Discussion

This study provides new insights to the mechanisms that allow animals to respond appropriately to diverse stresses. While HIF-1 and SKN-1 are both stress responsive transcription factors, they have distinct functions. For example, *skn-1*-deficient animals are less able to survive exposure to peroxide, while *hif-1-deficient* mutants are relatively resistant to this oxidizing agent [40] (Table 3). Conversely, while a deletion mutation in *hif-1* dramatically impairs survival in 0.5% oxygen [25], *skn-1* RNAi has little effect (Table 2). Thus, animals may benefit from cross-talk between these two transcription factors as they are challenged by oxygen deprivation and oxidative stress. Here, we provide evidence that SKN-1 promotes *egl-9* expression, thereby attenuating HIF-1 function.

### SKN-1 promotes *egl-9* expression, thereby inhibiting HIF-1

Our data support a model in which SKN-1 binds directly to the *egl-9* promoter to increase *egl-9* expression. This conclusion is further substantiated by the genome wide chromatin immunoprecipitation experiments that determined that SKN-1::GFP associated with DNA sequences in the *egl-9* 5’ regulatory region [47]. EGL-9 functions as a cellular oxygen sensor, and it mediates oxygen-dependent degradation of HIF-1 [26]. Hence, *skn-1* RNAi results in increased HIF-1 protein levels (Fig 2B). We expect that this regulatory interaction, in which SKN-1 can quickly down-regulate HIF-1, would allow animals to adapt quickly to changes in cellular conditions.

Prior studies have identified genes that are positively regulated by either SKN-1 or HIF-1, and these gene lists are largely non-overlapping [28, 32, 48–50]. Genes that are commonly regulated by both SKN-1 and HIF-1 include K10H10.2/*cysl-2*, F57B9.1, M05D6.5, and *rhy-1*. Two lines of evidence suggest that *rhy-1* may be a direct target of SKN-1. First, Oliveira et al. (2009) identified a potential SKN-1 binding site in the *rhy-1* promoter region. Second, the modENCODE project found that *rhy-1* 5’ regulatory sequences associated with SKN-1 *in vivo* [47]. Like *egl-9*, *rhy-1* is also a negative regulator of HIF-1 [28, 29]. Collectively, these data suggest that SKN-1 may act through both *egl-9* and *rhy-1* to reduce HIF-1 function. SKN-1 may also act through other pathways including the proteasomal pathway to influence the expression of HIF-1 targets. The RNAi screen reported here demonstrated that proteasomal dysfunction can increase the expression of the *Pnhr-57*::GFP reporter. Prior studies have shown that *skn-1* regulates several protesome components [32, 42, 47, 51–53], including six of the genes identified in the *Pnhr-57*::GFP RNAi screen (*rpn-11*, *rpt-5*, *rpt-6*, *pas-4*, *pbs-5*, and *pbs-6*).

Interactions between HIF-1 and SKN-1 are likely to be different in select cells, developmental stages, or environmental contexts. HIF-1, EGL-9 and SKN-1 each have other developmental functions, and some of these are specific to certain cell types [34, 54–58]. The downstream effects of SKN-1 or HIF-1 activation are further influenced by cellular or environmental contexts. For example, *C*. *elegans* SKN-1 can be activated by arsenite or by t-butyl hydroperoxide, but only a subset of SKN-1 targets are activated by either toxicant [32]. Also, while *C*. *elegans* SKN-1 and HIF-1 have distinct roles in peroxide and hypoxia stress responses, their functions overlap in selenium and hydrogen sulfide stress responses [13, 15, 35–37].

Similarly, the mammalian NRF and HIF transcription factors are very sensitive to environmental and physiological cues or stresses, and their regulatory relationships are context specific. Ischemia causes tissue hypoxia, which stabilizes HIF transcription factors. Once the ischemic tissue is reperfused, HIF transcription factors are degraded quickly and NRF2 is up-regulated, presumably to limit the oxidative damage [4, 5]. However, intermittent hypoxia has been shown to induce both HIF-1α and NRF2 [6, 7]. While NRF2 signaling activates HIF-1 in several cancer types [12], studies of the anti-inflammatory drug andrographolide in endothelial cells revealed interactions between NRF2 and the PHDs that modulate HIF-1 [59]. NRF2 and the HIF transcription factors have key roles in angiogenesis and iron regulation, and their functions can converge on developmental processes or feedback loops that modulate their activities [12, 60–62].

While the studies presented here illuminate key regulatory networks that govern stress response, they also point to outstanding questions. Given that HIF-1 is subject to oxygen-dependent degradation, why are *hif-1* mutants more resistant to oxidative stress? Prior studies provide some insight. Angeles-Albores et al. [63] showed that the genes up-regulated in *hif-1* mutants included genes involved in detoxification and stress response, such as glutathione S-transferases and cytochrome P450 CYP2 family enzymes. Hence, while *C*. *elegans* cannot adapt quickly to hypoxia in the absence of a functional *hif-1* gene [25], the *hif-1* mutation also causes widespread changes in gene expression that protect the animals from other insults, such as peroxide treatment. Future studies will further explore the ways in which stress response networks adapt to genetic or environmental changes and the impacts of these changes on organismal health.

### *Pnhr-57*::*GFP* as a marker for hypoxia-induced gene expression: Discoveries and insights from a genome-wide screen

Prior studies have demonstrated that *nhr-57* was induced by hypoxia in a *hif-1*-dependent manner and that over-expression of *Pnhr-57*::*GFP* in *egl-9* mutants required *hif-1* [13, 27–30]. Moreover, Bellier *et al*. (2009) found that HIF-1-mediated induction of *nhr-57* helped to protect *C*. *elegans* from the lethal effects of pore-forming toxins [64]. While these studies showed that *nhr-57* is a direct target of HIF-1, other transcription factors must also contribute to its expression. The studies presented here show that, while genes such as *egl-9* or *rhy-1* clearly regulate HIF-1 to control *Pnhr-57*::*GFP* expression, many other RNAi treatments can activate *Pnhr-57*::*GFP* through *hif-1*-independent pathways. The *Pnhr-57*::*GFP* reporter will continue to be a valuable marker, but these data inform our interpretations of studies that employ this reporter. While *Pnhr-57*::*GFP* is clearly regulated by HIF-1, it is important to compare expression of the reporter in wild-type and *hif-1*-deficient animals before drawing conclusions about HIF-1 activity.

We found that diverse RNAi treatments that compromise metabolic function or protein homeostasis increased *Pnhr-57*::*GFP* expression, and this effect did not require a functional *hif-1* gene. Interestingly, many of the genes integral to these processes have been shown to have roles in stress response and aging [65–70]. RNAi-mediated depletion of proteasomal components has also been shown to impact resistance to polyglutamine toxicity and to induce expression of *Pgpdh-1*::*GFP*, a marker for osmotic stress and glycerol production [71, 72].

Further characterization of the 179 RNAi treatments that increased *Pnhr-57*::GFP identified 13 genes that had much stronger effects in animals carrying a wild-type *hif-1* gene. These genes included *vhl-1*, *egl-9*, and *rhy-1*. These three genes had all been identified in prior studies as negative regulators of HIF-1 [26, 29]. The succinate dehydrogenase subunit *sdhb-1* was also found to have *hif-1*-dependent effects. This is especially interesting, since studies in cancer cell lines have shown that succinate can inhibit the enzymatic activities of HIF prolyl hydroxylases [73, 74]. *sams-1* and *sbp-1* encode the *C*. *elegans* S-adenosyl methionine synthetase and the SREBP homologs, respectively. The RNAi treatments of *sams-1*and *sbp-1*have a lesser impact on *Pnhr-57*::GFP levels in *hif-1* mutants, suggesting that the effects of *sams-1* and *sbp-1* RNAi on the reporter are mediated by HIF-1 (S5 Fig). Both of these genes have key roles in methionine metabolism and fatty acid biosynthesis [75], and it will be interesting to investigate the ways in which these important processes intersect with hypoxia response.

## Materials and methods

### Strains

The following strains were used in this study: wild-type N2 Bristol; ZG430: *Pnhr-57*::*GFP(iaIs07)*IV; *egl-9(sa307)*V; *hif-1(ia04)*V; *Phif-1*::*hif-1a*::*Myc*::*HA (iaIs28)*; ZG120: *Pnhr-57*::*GFP(iaIs07)*IV; ZG509: *rrf-3(pk1426)*II*; Pnhr-57*::*GFP(iaIs07)*IV; ZG508: *rrf-3(pk1426)*II; *Pnhr-57*::*GFP(iaIs07)*IV; *hif-1(ia04)*V; ZG429: *hif-1(ia04)*V; *Phif-1*::*hif-1a*::*Myc*::*HA(iaIs28)*; ZG472: *hif-1(ia04)*V; *Pegl-9*::*GFP(iaEx84);* ZG487: *hif-1(ia04)*V; *P(m)egl-9*::*GFP(iaEx96);* ZG488: *skn-1(zu67)*IV; *hif-1(ia04)*V; *Pegl-9*::*GFP(iaEx84)*. The transgenes expressing epitope-tagged HIF-1 protein were described and characterized previously [40]. The *skn-1 (zu67)* allele introduces a premature stop codon affecting *skn-1* mRNA isoforms a and c (https://wormbase.org/species/c_elegans/gene/WBGene00004804#0-9f-10).

### RNAi experiments

The RNAi screen was conducted as previously described [76], with few modifications. Each bacterial clone (expressing double-stranded RNA for one gene) was cultured in L-broth with 50 ug/mL ampicillin and 12.5 ug/mL tetracycline overnight at 37°C. The following morning, the bacteria were inoculated into new L-broth with 100 ug/mL ampicillin for 6 hours at 37°C before seeding on 24-well NGM agar plates with 25 ug/mL carbenicillin and 2 mM IPTG. Each RNAi clone was plated in duplicate. The following day, 15–25 L1-stage worms were added to each well. The plates were incubated at 15°C for 5–6 days, and then the worms were screened for positive *Pnhr-57*::GFP green fluorescence by stereomicroscopy. For the initial screen, 16,265 RNAi clones were assayed. Bacterial RNAi clones that increased the reporter were rescreened in two independent replicates, and the plasmid inserts were validated by sequencing.

For *skn-1* RNAi, N2 young adults (one day after L4 molt) were put on RNAi plates to lay eggs. *skn-1* RNAi causes maternal-effect lethality, in this study we examined the effects of first generation *skn-1* RNAi. Dead egg percentages given by the first generation *skn-1* RNAi adults were measured to check the *skn-1* RNAi efficiency. We routinely achieved as high as 90% dead egg percentages from the first generation *skn-1* RNAi adults, indicating high *skn-1* RNAi efficiency. While the *skn-1* and *sknr-1* genes are related, they are distinct enough to be differentially targeted by RNAi. In the screen reported here, *skn-1* RNAi altered *Pnhr-57*::GFP function, while *sknr-1* RNAi did not.

For *gsk-3* RNAi, N2 L4-stage worms were put on *gsk-3* RNAi plates or control RNAi plates to lay eggs. L4-stage progeny worms were sampled for western blot assays.

### Gene function annotation and function enrichment analyses

The DAVID (The Database for Annotation, Visualization and Integrated Discovery) tools (https://david.ncifcrf.gov) were used to annotate the 179 genes which increased *Pnhr-57*::GFP expression when knocked-down. These analyses identified the biological functions enriched among *Pnhr-57*::GFP regulators.

### Hypoxia and oxidative stress assays

To assess the relative effects of t-butyl-peroxide exposure, animals in the first day of adulthood were placed on NGM plates containing 7.5 mM t-butyl-peroxide, in the presence of bacterial food. The survival was scored after treating the animals for 6, 8 or 10 hours.

For hypoxia experiments, adults were allowed to lay eggs on standard NGM plates with OP50 bacterial food for 2 hours. The adults were then removed, and the plates with embryos were placed in a sealed plexiglass chamber with constant hypoxic gas flow at 21°C for 24 hours. Compressed air and 100% nitrogen were mixed to achieve 0.5% oxygen, and gas flow was controlled by an oxygen sensor [28]. After 24 hours, the plates were removed from the hypoxia chamber, and the un-hatched eggs were counted immediately. The plates were then maintained in room air (21°C). The adult worms were counted 72 hours after the eggs had been laid. Wild-type control animals hatched within 24 hours and reached adulthood within 72 hours.

### *Pegl-9*::*GFP* expression constructs

To generate the *Pegl-9*::*GFP* construct, a fragment that contained 1.6 kb of sequence upstream of the initiation ATG of *egl-9* gene was amplified by PCR using the forward primer 5’-CGCGCATGCGTGTATGTGTGTGAAAGAG-3’ and the reverse primer 5’-GCGGTCGACGCAACTTTTTTCTGTCACATTCAG-3’. The PCR product was cloned into the green fluorescence protein (GFP) vector pPD95.75 (gift from Andrew Fire). To create the *P(m)egl-9*::*GFP* point mutation construct, the predicted SKN-1 binding site TTTGTCAT [34, 77]was altered to CGACGGGC. Transgenic animals were generated by injection of DNA into the gonadal syncitium, using standard methods with *rol-6* (pRF4) as the co-injection marker [78]. For each construct, two independent transgenic lines were generated and assayed. For DIC and GFP imaging, animals were partially immobilized with sodium azide (10 mM). Sodium azide concentrations and duration were minimized to limit added stress to the worms.

### Protein blots

We performed pilot experiments to find the linear range for each western blot assay. To assay the expression of GFP or HIF-1 proteins, 20–100 L4-stage worms were collected and boiled for 5 min in 1X SDS sample buffer, and the lysates were size fractionated on polyacrylamide gels and analyzed by Western blots. The GFP-specific mouse monoclonal antibody (from Roche) was used at 1:500. The HA-specific mouse monoclonal antibody (from Cell Signaling) was used at 1:250. The secondary antibody (goat anti-mouse IgG+IgM from Biorad) was used at 1:2000 dilutions. The western blot images were analyzed by the Image J software. For each assay, three to five independent biological replicates were included.

### RNA extraction and real-time quantitative RT-PCR

Total RNA was isolated from synchronized L4-stage animals using Trizol (Invitrogen) and RNeasy Mini Kit (Qiagen). After being treated by RNase free DNase (Promega), total RNA was reverse transcribed to complementary DNA using Oligo dT_18_ primer and AffinityScript reverse transcriptase (Stratagene). Real-time quantitative PCR was performed using the iQ SYBR GREEN supermix (Bio-Rad) and Stratagene Mx4000 multiplex PCR system. In the assay, three biological replicates were included. And for each sample, three technical replicates were performed and they gave similar C_t_ values. And cDNA from 62.5 ng of total RNA was added to each PCR reaction. Relative mRNA quantification was performed using the efficiency-corrected comparative quantification method [79]. *inf-1*, a gene not regulated by hypoxia, was used as the reference gene [28]. The primer sequences for *inf-1* real-time quantitative PCR are included in S1 Fig. The primers for *egl-9* real-time quantitative PCR are the forward primer 5’-GCCGACTTTCAATCCACTTC-3’ and reverse primer 5’- AATGATCGGAGATCGACTGG-3’. There are seven isoforms of *egl-9* mRNA transcripts (https://wormbase.org/species/c_elegans/gene/WBGene00001178#0-9f-10). This primer set can detect six out of seven *egl-9* mRNA isoforms. The isoform d.1 will not be detected by this primer set, because the forward primer is located within the eighth intron of the unspliced isoform d.1.

### Statistical analyses

All the experiments for testing the hypothesis that *skn-1* transcriptionally regulates *egl-9* followed a randomized complete block design. All of these experiments were analyzed with an ANOVA model and an F-test was conducted. Briefly, to assay the simple effect of *skn-1* RNAi treatment on *Pnhr-57*::GFP at each hypoxia time point, Log2 transformed western blot intensities of *Pnhr-57*::GFP were analyzed with hypoxia time (2h, 4h, or 6h, treated as a categorical variable), RNAi treatment (*skn-1* RNAi or control RNAi), hypoxia time by RNAi treatment interaction, and the three independently replicated experiments treated as fixed effect factors, assuming normality and homoscedasticity of errors. *skn-1(Zu67)* effect on *Pegl-9*::GFP heat shock induction, and heat shock or *gsk-3* RNAi effect on *Pegl-9*::GFP and *P(m)egl-9*::GFP induction were assayed using the similar ANOVA model as that for *Pnhr-57*::GFP. Except that Log2 fold changes were analyzed. The fold change was determined by dividing the western blot intensity of the heat shock (or *gsk-3* RNAi) sample by the corresponding non-heat shock (or control RNAi) sample. Similarly, to assay the simple effect of *skn-1* RNAi treatment on *egl-9* mRNA expressions at each hypoxia time point, Log2 comparative expression (compared to *inf-1*) were analyzed using the same ANOVA model as that for *Pnhr-57*::GFP, except that error variances are assumed to be different for different hypoxia time. Accordingly, tests of interesting linear contrasts employed Satterthwaite type approximation to the degrees of freedom.

## Supporting information

S1 Fig *nhr-57* promoter HIF-1 chromatin immunoprecipitation experiments. (A) *nhr-57* promoter sequence and positions of primers used for real-time quantitative PCR assays in HIF-1 chromatin immunoprecipitation experiments. (B) Primer sequences for real-time quantitative PCR assays in *nhr-57* promoter HIF-1 chromatin immunoprecipitation experiments. (C) Chromatin co-immunoprecipitation data. In these experiments the endogenous *hif-1* locus was disrupted by the *ia04* large deletion and HIF-1 function was restored by the *Phif-1*::*hif-1a*::*Myc*::*HA* transgene [40]. The relative amounts of *nhr-57* promoter regions that co-immunoprecipitated with HIF-1::Myc::HA was determined by real-time quantitative PCR. The bars show the average enriched fold from at least three independent replicates. *inf-1*, a gene not regulated by HIF-1, was used as the reference gene.
(DOCX)

S2 Fig *Pegl-9*::*GFP* expression in L1, 2, 3 and adult-stage animals under normal culture conditions and heat shock. (PDF)

S3 Fig Heat shock induced another independent *Pegl-9*::*GFP* transgenic line in animals carrying the wild-type s*kn-1* allele, but did not induce the reporter in animals carrying the *skn-1(zu67)* loss-of-function mutation. (PDF)

S4 Fig Heat shock increased the expression of *Pegl-9*::*GFP*, but did not increase the expression of the reporter in which the putative SKN-1 binding site was mutated (*P(m)egl-9*::*GFP*) in another independent line. (PDF)

S5 Fig RNAi inactivation of *sams-1* or *sbp-1* increased *Pnhr-57*::*GFP* expression. (A) RNAi for *sams-1* (S-adenosyl methionine synthetase) increased *Pnhr-57*::*GFP* expression more than 7-fold in animals carrying the wild-type *hif-1* allele relative to control RNAi, and increased the reporter 3-fold in animals carrying the *hif-1(ia04)* deletion. The difference in RNAi effect between *hif-1(+)* and *hif-1(ia04)* strains is statistically significant (*p < 0.05, from six independent experiments, by student *t-*test). (B) RNAi for the SREBP homolog *sbp-1* increased expression of the reporter more than 3-fold in animals carrying the wild-type *hif-1* allele, but had no effect on *Pnhr-57*::*GFP* expression in *hif-1(ia04)* mutants. The difference in RNAi effect between *hif-1(+)* and *hif-1(ia04)* strains is statistically significant (*p < 0.05, from five independent experiments, by student *t-*test). GFP levels were determined by protein blots, and the control animals were fed on bacteria carrying the empty RNAi vector (L4440). The experiments were conducted in RNAi-sensitive strains (*rrf-3(pk1426*)).
(DOCX)

S1 Table Genes increased *Pnhr-57*::*GFP* expression when knocked-down by RNAi. (XLSX)

S2 Table Genes for which RNAi caused *hif-1*-dependent increase of *Pnhr-57*::*GFP* expression. (DOCX)

S1 Raw images (PDF)

## References

[1] Beckman KB, Ames BN. The free radical theory of aging matures. Physiol Rev. 1998;78(2):547–81. Epub 1998/04/30. doi: 10.1152/physrev.1998.78.2.547 . 9562038

[2] Finkel T. Oxidant signals and oxidative stress. Current opinion in cell biology. 2003;15(2):247–54. Epub 2003/03/22. doi: 10.1016/s0955-0674(03)00002-4 . 12648682

[3] Finkel T, Holbrook NJ. Oxidants, oxidative stress and the biology of ageing. Nature. 2000;408(6809):239–47. Epub 2000/11/23. doi: 10.1038/35041687 . 11089981

[4] Kim YJ, Ahn JY, Liang P, Ip C, Zhang Y, Park YM. Human prx1 gene is a target of Nrf2 and is up-regulated by hypoxia/reoxygenation: implication to tumor biology. Cancer Res. 2007;67(2):546–54. Epub 2007/01/20. 67/2/546 [pii] doi: 10.1158/0008-5472.CAN-06-2401 . 17234762

[5] Leonard MO, Kieran NE, Howell K, Burne MJ, Varadarajan R, Dhakshinamoorthy S, et al. Reoxygenation-specific activation of the antioxidant transcription factor Nrf2 mediates cytoprotective gene expression in ischemia-reperfusion injury. FASEB J. 2006;20(14):2624–6. Epub 2006/12/05. doi: 10.1096/fj.06-5097fje . 17142801

[6] Malec V, Gottschald OR, Li S, Rose F, Seeger W, Hanze J. HIF-1 alpha signaling is augmented during intermittent hypoxia by induction of the Nrf2 pathway in NOX1-expressing adenocarcinoma A549 cells. Free Radic Biol Med. 2010;48(12):1626–35. Epub 2010/03/30. doi: 10.1016/j.freeradbiomed.2010.03.008 . 20347035

[7] Martinive P, Defresne F, Bouzin C, Saliez J, Lair F, Gregoire V, et al. Preconditioning of the tumor vasculature and tumor cells by intermittent hypoxia: implications for anticancer therapies. Cancer Res. 2006;66(24):11736–44. doi: 10.1158/0008-5472.CAN-06-2056 . 17178869

[8] Thimmulappa RK, Mai KH, Srisuma S, Kensler TW, Yamamoto M, Biswal S. Identification of Nrf2-regulated genes induced by the chemopreventive agent sulforaphane by oligonucleotide microarray. Cancer Res. 2002;62(18):5196–203. Epub 2002/09/18. . 12234984

[9] Hayes JD, McMahon M. Molecular basis for the contribution of the antioxidant responsive element to cancer chemoprevention. Cancer Lett. 2001;174(2):103–13. Epub 2001/11/02. S0304383501006954 [pii]. doi: 10.1016/s0304-3835(01)00695-4 . 11689285

[10] Hough RB, Piatigorsky J. Preferential transcription of rabbit Aldh1a1 in the cornea: implication of hypoxia-related pathways. Molecular and cellular biology. 2004;24(3):1324–40. Epub 2004/01/20. doi: 10.1128/MCB.24.3.1324-1340.2004 ; PubMed Central PMCID: PMC321433. 14729976 PMC321433

[11] Yeligar SM, Machida K, Kalra VK. Ethanol-induced HO-1 and NQO1 are differentially regulated by HIF-1alpha and Nrf2 to attenuate inflammatory cytokine expression. J Biol Chem. 2010;285(46):35359–73. Epub 2010/09/14. doi: 10.1074/jbc.M110.138636 ; PubMed Central PMCID: PMC2975160. 20833713 PMC2975160

[12] Toth RK, Warfel NA. Strange Bedfellows: Nuclear Factor, Erythroid 2-Like 2 (Nrf2) and Hypoxia-Inducible Factor 1 (HIF-1) in Tumor Hypoxia. Antioxidants (Basel). 2017;6(2). doi: 10.3390/antiox6020027 ; PubMed Central PMCID: PMC5488007. 28383481 PMC5488007

[13] Budde MW, Roth MB. Hydrogen sulfide increases hypoxia-inducible factor-1 activity independently of von Hippel-Lindau tumor suppressor-1 in C. elegans. Mol Biol Cell. 2010;21(1):212–7. Epub 2009/11/06. E09-03-0199 [pii] doi: 10.1091/mbc.e09-03-0199 ; PubMed Central PMCID: PMC2801715. 19889840 PMC2801715

[14] Powell-Coffman JA. Hypoxia signaling and resistance in C. elegans. Trends Endocrinol Metab. 2010;21(7):435–40. Epub 2010/03/26. S1043-2760(10)00041-X [pii] doi: 10.1016/j.tem.2010.02.006 20335046

[15] Romanelli-Credrez L, Doitsidou M, Alkema MJ, Salinas G. HIF-1 Has a Central Role in Caenorhabditis elegans Organismal Response to Selenium. Front Genet. 2020;11:63. doi: 10.3389/fgene.2020.00063 ; PubMed Central PMCID: PMC7052493. 32161616 PMC7052493

[16] Saldanha JN, Parashar A, Pandey S, Powell-Coffman JA. Multiparameter behavioral analyses provide insights to mechanisms of cyanide resistance in Caenorhabditis elegans. Toxicol Sci. 2013;135(1):156–68. doi: 10.1093/toxsci/kft138 ; PubMed Central PMCID: PMC3748764. 23805000 PMC3748764

[17] Shao Z, Zhang Y, Ye Q, Saldanha JN, Powell-Coffman JA. C. elegans SWAN-1 Binds to EGL-9 and regulates HIF-1-mediated resistance to the bacterial pathogen Pseudomonas aeruginosa PAO1. PLoS Pathog. 2010;6(8):e1001075. Epub 2010/09/25. doi: 10.1371/journal.ppat.1001075 ; PubMed Central PMCID: PMC2928816. 20865124 PMC2928816

[18] Kaelin WG Jr., Ratcliffe PJ. Oxygen sensing by metazoans: the central role of the HIF hydroxylase pathway. Mol Cell. 2008;30(4):393–402. Epub 2008/05/24. S1097-2765(08)00292-X [pii] doi: 10.1016/j.molcel.2008.04.009 . 18498744

[19] Semenza GL. Oxygen sensing, homeostasis, and disease. N Engl J Med. 2011;365(6):537–47. doi: 10.1056/NEJMra1011165 . 21830968

[20] Wang GL, Jiang BH, Rue EA, Semenza GL. Hypoxia-inducible factor 1 is a basic-helix-loop-helix-PAS heterodimer regulated by cellular O2 tension. Proc Natl Acad Sci U S A. 1995;92(12):5510–4. Epub 1995/06/06. doi: 10.1073/pnas.92.12.5510 ; PubMed Central PMCID: PMC41725. 7539918 PMC41725

[21] Maxwell PH, Wiesener MS, Chang GW, Clifford SC, Vaux EC, Cockman ME, et al. The tumour suppressor protein VHL targets hypoxia-inducible factors for oxygen-dependent proteolysis. Nature. 1999;399(6733):271–5. doi: 10.1038/20459 . 10353251

[22] Jaakkola P, Mole DR, Tian YM, Wilson MI, Gielbert J, Gaskell SJ, et al. Targeting of HIF-alpha to the von Hippel-Lindau ubiquitylation complex by O2-regulated prolyl hydroxylation. Science. 2001;292(5516):468–72. doi: 10.1126/science.1059796 . 11292861

[23] Ivan M, Kondo K, Yang H, Kim W, Valiando J, Ohh M, et al. HIFalpha targeted for VHL-mediated destruction by proline hydroxylation: implications for O2 sensing. Science. 2001;292(5516):464–8. doi: 10.1126/science.1059817 . 11292862

[24] Powell-Coffman JA, Bradfield CA, Wood WB. Caenorhabditis elegans orthologs of the aryl hydrocarbon receptor and its heterodimerization partner the aryl hydrocarbon receptor nuclear translocator. Proc Natl Acad Sci U S A. 1998;95(6):2844–9. Epub 1998/04/18. doi: 10.1073/pnas.95.6.2844 . 9501178 PMC19657

[25] Jiang H, Guo R, Powell-Coffman JA. The Caenorhabditis elegans hif-1 gene encodes a bHLH-PAS protein that is required for adaptation to hypoxia. Proc Natl Acad Sci U S A. 2001;98(14):7916–21. Epub 2001/06/28. doi: 10.1073/pnas.141234698 [pii]. ; PubMed Central PMCID: PMC35443. 11427734 PMC35443

[26] Epstein AC, Gleadle JM, McNeill LA, Hewitson KS, O’Rourke J, Mole DR, et al. C. elegans EGL-9 and mammalian homologs define a family of dioxygenases that regulate HIF by prolyl hydroxylation. Cell. 2001;107(1):43–54. Epub 2001/10/12. S0092-8674(01)00507-4 [pii]. doi: 10.1016/s0092-8674(01)00507-4 . 11595184

[27] Bishop T, Lau KW, Epstein AC, Kim SK, Jiang M, O’Rourke D, et al. Genetic analysis of pathways regulated by the von Hippel-Lindau tumor suppressor in Caenorhabditis elegans. PLoS Biol. 2004;2(10):e289. Epub 2004/09/14. doi: 10.1371/journal.pbio.0020289 . 15361934 PMC515368

[28] Shen C, Nettleton D, Jiang M, Kim SK, Powell-Coffman JA. Roles of the HIF-1 hypoxia-inducible factor during hypoxia response in Caenorhabditis elegans. J Biol Chem. 2005;280(21):20580–8. Epub 2005/03/23. M501894200 [pii] doi: 10.1074/jbc.M501894200 . 15781453

[29] Shen C, Shao Z, Powell-Coffman JA. The Caenorhabditis elegans rhy-1 gene inhibits HIF-1 hypoxia-inducible factor activity in a negative feedback loop that does not include vhl-1. Genetics. 2006;174(3):1205–14. Epub 2006/09/19. genetics.106.063594 [pii] doi: 10.1534/genetics.106.063594 . 16980385 PMC1667075

[30] Shao Z, Zhang Y, Powell-Coffman JA. Two distinct roles for EGL-9 in the regulation of HIF-1-mediated gene expression in Caenorhabditis elegans. Genetics. 2009;183(3):821–9. Epub 2009/09/10. genetics.109.107284 [pii] doi: 10.1534/genetics.109.107284 ; PubMed Central PMCID: PMC2778979. 19737748 PMC2778979

[31] Walker AK, See R, Batchelder C, Kophengnavong T, Gronniger JT, Shi Y, et al. A conserved transcription motif suggesting functional parallels between Caenorhabditis elegans SKN-1 and Cap’n’Collar-related basic leucine zipper proteins. J Biol Chem. 2000;275(29):22166–71. Epub 2000/04/15. doi: 10.1074/jbc.M001746200 [pii]. . 10764775

[32] Oliveira RP, Abate JP, Dilks K, Landis J, Ashraf J, Murphy CT, et al. Condition-adapted stress and longevity gene regulation by Caenorhabditis elegans SKN-1/Nrf. Aging Cell. 2009;8(5):524–41. Epub 2009/07/07. ACE501 [pii] doi: 10.1111/j.1474-9726.2009.00501.x . 19575768 PMC2776707

[33] Park SK, Tedesco PM, Johnson TE. Oxidative stress and longevity in Caenorhabditis elegans as mediated by SKN-1. Aging Cell. 2009;8(3):258–69. Epub 2009/07/25. doi: 10.1111/j.1474-9726.2009.00473.x ; PubMed Central PMCID: PMC2762118. 19627265 PMC2762118

[34] An JH, Blackwell TK. SKN-1 links C. elegans mesendodermal specification to a conserved oxidative stress response. Genes Dev. 2003;17(15):1882–93. Epub 2003/07/19. doi: 10.1101/gad.1107803 . 12869585 PMC196237

[35] Horsman JW, Heinis FI, Miller DL. A Novel Mechanism To Prevent H2S Toxicity in Caenorhabditis elegans. Genetics. 2019;213(2):481–90. doi: 10.1534/genetics.119.302326 ; PubMed Central PMCID: PMC6781907. 31371406 PMC6781907

[36] Li WH, Chang CH, Huang CW, Wei CC, Liao VH. Selenite enhances immune response against Pseudomonas aeruginosa PA14 via SKN-1 in Caenorhabditis elegans. PLoS One. 2014;9(8):e105810. doi: 10.1371/journal.pone.0105810 ; PubMed Central PMCID: PMC4141825. 25147937 PMC4141825

[37] Miller DL, Budde MW, Roth MB. HIF-1 and SKN-1 coordinate the transcriptional response to hydrogen sulfide in Caenorhabditis elegans. PLoS One. 2011;6(9):e25476. Epub 2011/10/08. doi: 10.1371/journal.pone.0025476 [pii]. ; PubMed Central PMCID: PMC3183046. 21980473 PMC3183046

[38] Kamath RS, Ahringer J. Genome-wide RNAi screening in Caenorhabditis elegans. Methods. 2003;30(4):313–21. Epub 2003/06/28. S1046202303000501 [pii]. doi: 10.1016/s1046-2023(03)00050-1 . 12828945

[39] Inoue H, Hisamoto N, An JH, Oliveira RP, Nishida E, Blackwell TK, et al. The C. elegans p38 MAPK pathway regulates nuclear localization of the transcription factor SKN-1 in oxidative stress response. Genes Dev. 2005;19(19):2278–83. Epub 2005/09/17. gad.1324805 [pii] doi: 10.1101/gad.1324805 . 16166371 PMC1240035

[40] Zhang Y, Shao Z, Zhai Z, Shen C, Powell-Coffman JA. The HIF-1 hypoxia-inducible factor modulates lifespan in C. elegans. PLoS One. 2009;4(7):e6348. Epub 2009/07/28. doi: 10.1371/journal.pone.0006348 . 19633713 PMC2711329

[41] Padilla PA, Nystul TG, Zager RA, Johnson AC, Roth MB. Dephosphorylation of cell cycle-regulated proteins correlates with anoxia-induced suspended animation in Caenorhabditis elegans. Mol Biol Cell. 2002;13(5):1473–83. Epub 2002/05/15. doi: 10.1091/mbc.01-12-0594 . 12006646 PMC111120

[42] Li X, Matilainen O, Jin C, Glover-Cutter KM, Holmberg CI, Blackwell TK. Specific SKN-1/Nrf stress responses to perturbations in translation elongation and proteasome activity. PLoS Genet. 2011;7(6):e1002119. Epub 2011/06/23. doi: 10.1371/journal.pgen.1002119 ; PubMed Central PMCID: PMC3111486. 21695230 PMC3111486

[43] Tullet JM, Hertweck M, An JH, Baker J, Hwang JY, Liu S, et al. Direct inhibition of the longevity-promoting factor SKN-1 by insulin-like signaling in C. elegans. Cell. 2008;132(6):1025–38. Epub 2008/03/25. S0092-8674(08)00130-X [pii] doi: 10.1016/j.cell.2008.01.030 . 18358814 PMC2367249

[44] Wang J, Robida-Stubbs S, Tullet JM, Rual JF, Vidal M, Blackwell TK. RNAi screening implicates a SKN-1-dependent transcriptional response in stress resistance and longevity deriving from translation inhibition. PLoS Genet. 2010;6(8). Epub 2010/08/12. doi: 10.1371/journal.pgen.1001048 ; PubMed Central PMCID: PMC2916858. 20700440 PMC2916858

[45] Darby C, Cosma CL, Thomas JH, Manoil C. Lethal paralysis of Caenorhabditis elegans by Pseudomonas aeruginosa. Proc Natl Acad Sci U S A. 1999;96(26):15202–7. Epub 1999/12/28. doi: 10.1073/pnas.96.26.15202 . 10611362 PMC24797

[46] An JH, Vranas K, Lucke M, Inoue H, Hisamoto N, Matsumoto K, et al. Regulation of the Caenorhabditis elegans oxidative stress defense protein SKN-1 by glycogen synthase kinase-3. Proc Natl Acad Sci U S A. 2005;102(45):16275–80. doi: 10.1073/pnas.0508105102 ; PubMed Central PMCID: PMC1283458. 16251270 PMC1283458

[47] Niu W, Lu ZJ, Zhong M, Sarov M, Murray JI, Brdlik CM, et al. Diverse transcription factor binding features revealed by genome-wide ChIP-seq in C. elegans. Genome research. 2011;21(2):245–54. Epub 2010/12/24. doi: 10.1101/gr.114587.110 ; PubMed Central PMCID: PMC3032928. 21177963 PMC3032928

[48] Glover-Cutter KM, Lin S, Blackwell TK. Integration of the unfolded protein and oxidative stress responses through SKN-1/Nrf. PLoS Genet. 2013;9(9):e1003701. doi: 10.1371/journal.pgen.1003701 ; PubMed Central PMCID: PMC3772064. 24068940 PMC3772064

[49] Paek J, Lo JY, Narasimhan SD, Nguyen TN, Glover-Cutter K, Robida-Stubbs S, et al. Mitochondrial SKN-1/Nrf mediates a conserved starvation response. Cell Metab. 2012;16(4):526–37. doi: 10.1016/j.cmet.2012.09.007 ; PubMed Central PMCID: PMC3774140. 23040073 PMC3774140

[50] Robida-Stubbs S, Glover-Cutter K, Lamming DW, Mizunuma M, Narasimhan SD, Neumann-Haefelin E, et al. TOR signaling and rapamycin influence longevity by regulating SKN-1/Nrf and DAF-16/FoxO. Cell Metab. 2012;15(5):713–24. doi: 10.1016/j.cmet.2012.04.007 ; PubMed Central PMCID: PMC3348514. 22560223 PMC3348514

[51] Chondrogianni N, Georgila K, Kourtis N, Tavernarakis N, Gonos ES. 20S proteasome activation promotes life span extension and resistance to proteotoxicity in Caenorhabditis elegans. FASEB J. 2015;29(2):611–22. doi: 10.1096/fj.14-252189 ; PubMed Central PMCID: PMC4314225. 25395451 PMC4314225

[52] Pickering AM, Staab TA, Tower J, Sieburth D, Davies KJ. A conserved role for the 20S proteasome and Nrf2 transcription factor in oxidative stress adaptation in mammals, Caenorhabditis elegans and Drosophila melanogaster. J Exp Biol. 2013;216(Pt 4):543–53. doi: 10.1242/jeb.074757 ; PubMed Central PMCID: PMC3561776. 23038734 PMC3561776

[53] Steinbaugh MJ, Narasimhan SD, Robida-Stubbs S, Moronetti Mazzeo LE, Dreyfuss JM, Hourihan JM, et al. Lipid-mediated regulation of SKN-1/Nrf in response to germ cell absence. Elife. 2015;4. doi: 10.7554/eLife.07836 ; PubMed Central PMCID: PMC4541496. 26196144 PMC4541496

[54] Bishop NA, Guarente L. Two neurons mediate diet-restriction-induced longevity in C. elegans. Nature. 2007;447(7144):545–9. Epub 2007/06/01. nature05904 [pii] doi: 10.1038/nature05904 . 17538612

[55] Chen D, Thomas EL, Kapahi P. HIF-1 modulates dietary restriction-mediated lifespan extension via IRE-1 in Caenorhabditis elegans. PLoS Genet. 2009;5(5):e1000486. Epub 2009/05/23. doi: 10.1371/journal.pgen.1000486 . 19461873 PMC2676694

[56] Park EC, Ghose P, Shao Z, Ye Q, Kang L, Xu XZ, et al. Hypoxia regulates glutamate receptor trafficking through an HIF-independent mechanism. EMBO J. 2012;31(6):1379–93. Epub 2012/01/19. doi: 10.1038/emboj.2011.499 ; PubMed Central PMCID: PMC3321172. 22252129 PMC3321172

[57] Pocock R, Hobert O. Oxygen levels affect axon guidance and neuronal migration in Caenorhabditis elegans. Nat Neurosci. 2008;11(8):894–900. Epub 2008/07/01. nn.2152 [pii] doi: 10.1038/nn.2152 . 18587389

[58] Chang AJ, Bargmann CI. Hypoxia and the HIF-1 transcriptional pathway reorganize a neuronal circuit for oxygen-dependent behavior in Caenorhabditis elegans. Proc Natl Acad Sci U S A. 2008;105(20):7321–6. doi: 10.1073/pnas.0802164105 ; PubMed Central PMCID: PMC2438248. 18477695 PMC2438248

[59] Lin HC, Su SL, Lu CY, Lin AH, Lin WC, Liu CS, et al. Andrographolide inhibits hypoxia-induced HIF-1alpha-driven endothelin 1 secretion by activating Nrf2/HO-1 and promoting the expression of prolyl hydroxylases 2/3 in human endothelial cells. Environ Toxicol. 2017;32(3):918–30. doi: 10.1002/tox.22293 . 27297870

[60] Duarte TL, Talbot NP, Drakesmith H. NRF2 and Hypoxia-Inducible Factors: Key Players in the Redox Control of Systemic Iron Homeostasis. Antioxid Redox Signal. 2020. doi: 10.1089/ars.2020.8148 . 32791852

[61] Li L, Pan H, Wang H, Li X, Bu X, Wang Q, et al. Interplay between VEGF and Nrf2 regulates angiogenesis due to intracranial venous hypertension. Sci Rep. 2016;6:37338. doi: 10.1038/srep37338 ; PubMed Central PMCID: PMC5116754. 27869147 PMC5116754

[62] Wong BW, Kuchnio A, Bruning U, Carmeliet P. Emerging novel functions of the oxygen-sensing prolyl hydroxylase domain enzymes. Trends Biochem Sci. 2013;38(1):3–11. doi: 10.1016/j.tibs.2012.10.004 . 23200187

[63] Angeles-Albores D, Puckett Robinson C, Williams BA, Wold BJ, Sternberg PW. Reconstructing a metazoan genetic pathway with transcriptome-wide epistasis measurements. Proc Natl Acad Sci U S A. 2018;115(13):E2930–E9. doi: 10.1073/pnas.1712387115 ; PubMed Central PMCID: PMC5879656. 29531064 PMC5879656

[64] Bellier A, Chen CS, Kao CY, Cinar HN, Aroian RV. Hypoxia and the hypoxic response pathway protect against pore-forming toxins in C. elegans. PLoS Pathog. 2009;5(12):e1000689. Epub 2009/12/17. doi: 10.1371/journal.ppat.1000689 . 20011506 PMC2785477

[65] Lee SS, Lee RY, Fraser AG, Kamath RS, Ahringer J, Ruvkun G. A systematic RNAi screen identifies a critical role for mitochondria in C. elegans longevity. Nat Genet. 2003;33(1):40–8. Epub 2002/11/26. doi: 10.1038/ng1056 [pii]. . 12447374

[66] Hansen M, Hsu AL, Dillin A, Kenyon C. New genes tied to endocrine, metabolic, and dietary regulation of lifespan from a Caenorhabditis elegans genomic RNAi screen. PLoS Genet. 2005;1(1):119–28. Epub 2005/08/17. doi: 10.1371/journal.pgen.0010017 ; PubMed Central PMCID: PMC1183531. 16103914 PMC1183531

[67] Hamilton B, Dong Y, Shindo M, Liu W, Odell I, Ruvkun G, et al. A systematic RNAi screen for longevity genes in C. elegans. Genes Dev. 2005;19(13):1544–55. Epub 2005/07/07. 19/13/1544 [pii] doi: 10.1101/gad.1308205 . 15998808 PMC1172061

[68] Curran SP, Ruvkun G. Lifespan regulation by evolutionarily conserved genes essential for viability. PLoS Genet. 2007;3(4):e56. Epub 2007/04/07. 07-PLGE-RA-0112R1 [pii] doi: 10.1371/journal.pgen.0030056 . 17411345 PMC1847696

[69] Kim Y, Sun H. Functional genomic approach to identify novel genes involved in the regulation of oxidative stress resistance and animal lifespan. Aging Cell. 2007;6(4):489–503. Epub 2007/07/05. ACE302 [pii] doi: 10.1111/j.1474-9726.2007.00302.x . 17608836

[70] Sugimoto T, Mori C, Takanami T, Sasagawa Y, Saito R, Ichiishi E, et al. Caenorhabditis elegans par2.1/mtssb-1 is essential for mitochondrial DNA replication and its defect causes comprehensive transcriptional alterations including a hypoxia response. Experimental cell research. 2008;314(1):103–14. Epub 2007/09/29. doi: 10.1016/j.yexcr.2007.08.015 . 17900564

[71] Lamitina T, Huang CG, Strange K. Genome-wide RNAi screening identifies protein damage as a regulator of osmoprotective gene expression. Proc Natl Acad Sci U S A. 2006;103(32):12173–8. Epub 2006/08/02. 0602987103 [pii] doi: 10.1073/pnas.0602987103 . 16880390 PMC1567714

[72] Nollen EA, Garcia SM, van Haaften G, Kim S, Chavez A, Morimoto RI, et al. Genome-wide RNA interference screen identifies previously undescribed regulators of polyglutamine aggregation. Proc Natl Acad Sci U S A. 2004;101(17):6403–8. Epub 2004/04/16. doi: 10.1073/pnas.0307697101 [pii]. . 15084750 PMC404057

[73] Selak MA, Armour SM, MacKenzie ED, Boulahbel H, Watson DG, Mansfield KD, et al. Succinate links TCA cycle dysfunction to oncogenesis by inhibiting HIF-alpha prolyl hydroxylase. Cancer Cell. 2005;7(1):77–85. Epub 2005/01/18. S153561080400368X [pii] doi: 10.1016/j.ccr.2004.11.022 . 15652751

[74] Koivunen P, Hirsila M, Remes AM, Hassinen IE, Kivirikko KI, Myllyharju J. Inhibition of hypoxia-inducible factor (HIF) hydroxylases by citric acid cycle intermediates: possible links between cell metabolism and stabilization of HIF. J Biol Chem. 2007;282(7):4524–32. Epub 2006/12/22. M610415200 [pii] doi: 10.1074/jbc.M610415200 . 17182618

[75] Walker AK, Jacobs RL, Watts JL, Rottiers V, Jiang K, Finnegan DM, et al. A conserved SREBP-1/phosphatidylcholine feedback circuit regulates lipogenesis in metazoans. Cell. 2011;147(4):840–52. Epub 2011/11/01. doi: 10.1016/j.cell.2011.09.045 ; PubMed Central PMCID: PMC3384509. 22035958 PMC3384509

[76] Simmer F, Moorman C, van der Linden AM, Kuijk E, van den Berghe PV, Kamath RS, et al. Genome-wide RNAi of C. elegans using the hypersensitive rrf-3 strain reveals novel gene functions. PLoS Biol. 2003;1(1):E12. Epub 2003/10/14. doi: 10.1371/journal.pbio.0000012 ; PubMed Central PMCID: PMC212692. 14551910 PMC212692

[77] Blackwell TK, Bowerman B, Priess JR, Weintraub H. Formation of a monomeric DNA binding domain by Skn-1 bZIP and homeodomain elements. Science. 1994;266(5185):621–8. Epub 1994/10/28. doi: 10.1126/science.7939715 . 7939715

[78] Mello CC, Kramer JM, Stinchcomb D, Ambros V. Efficient gene transfer in C.elegans: extrachromosomal maintenance and integration of transforming sequences. EMBO J. 1991;10(12):3959–70. Epub 1991/12/01. . 1935914 10.1002/j.1460-2075.1991.tb04966.x PMC453137

[79] Pfaffl MW. A new mathematical model for relative quantification in real-time RT-PCR. Nucleic Acids Res. 2001;29(9):e45. Epub 2001/05/09. doi: 10.1093/nar/29.9.e45 ; PubMed Central PMCID: PMC55695. 11328886 PMC55695

