# Supplementary material for: Crosstalk in oxygen homeostasis networks: SKN-1/NRF inhibits the HIF-1 hypoxia-inducible factor in Caenorhabditis elegans
Source: PLoS One. 2021 Jul 9;16(7):e0249103. doi: 10.1371/journal.pone.0249103 (PMC8270126; doi:10.1371/journal.pone.0249103)
Supplement: S2 Fig — (PDF) [file pone.0249103.s002.pdf]

S2 Fig. *Pegl-9::GFP* expression in L1, 2 ,3 and adult-stage animals under normal culture conditions and heat shock.

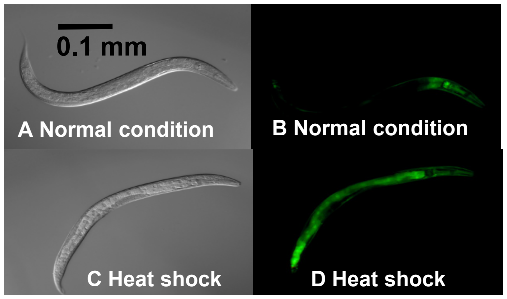

Heat shock alters *Pegl-9::GFP* expression in L1-stage worms. (A-D) *Pegl-9::GFP* expression in L1-stage animals under normal culture conditions and heat shock. Animals are shown as DIC images (A and C) and corresponding images of GFP fluorescence (B and D). (A and B) Under normal conditions, *Pegl-9::GFP* was expressed in the body muscle, vulva, pharynx, anterior intestine, rectal cells and additional cells in the tail. (C and D) After heat shock treatment (29°C for 20 hours), *Pegl-9::GFP* was strongly induced in the intestine.

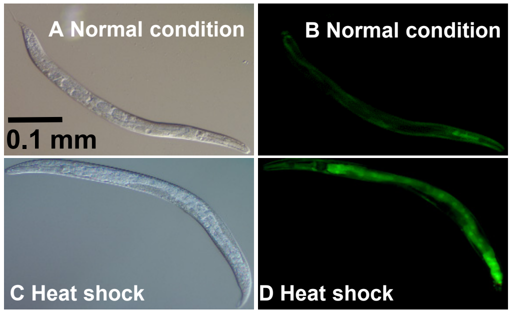

Heat shock alters *Pegl-9::GFP* expression in L2-stage worms. (A-D) *Pegl-9::GFP* expression in L2-stage animals under normal culture conditions and heat shock. Animals are shown as DIC images (A and C) and corresponding images of GFP fluorescence (B and D). (A and B) Under normal conditions, *Pegl-9::GFP* was expressed in the body muscle, vulva, pharynx, anterior intestine, rectal cells and additional cells in the tail. (C and D) After heat shock treatment (29°C for 20 hours), *Pegl-9::GFP* was strongly induced in the intestine.

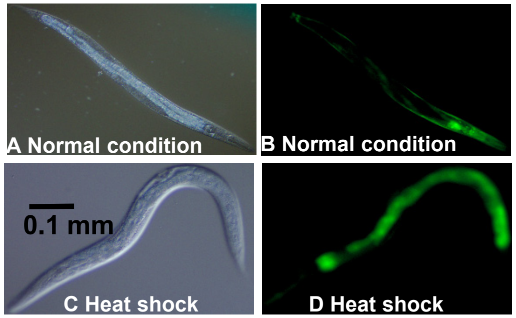

Heat shock alters *Pegl-9::GFP* expression in L3-stage worms. (A-D) *Pegl-9::GFP* expression in L3-stage animals under normal culture conditions and heat shock. Animals are shown as DIC images (A and C) and corresponding images of GFP fluorescence (B and D). (A and B) Under normal conditions, *Pegl-9::GFP* was expressed in the body muscle, vulva, pharynx, anterior intestine, rectal cells and additional cells in the tail. (C and D) After heat shock treatment (29°C for 20 hours), *Pegl-9::GFP* was strongly induced in the intestine.

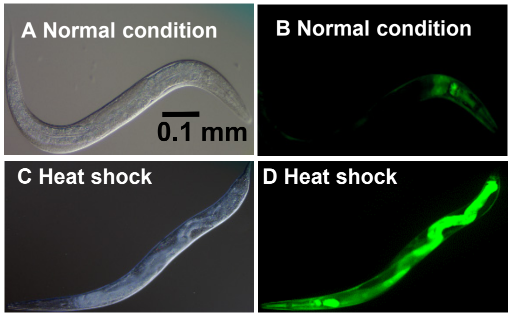

Heat shock alters *Pegl-9::GFP* expression in adult worms. (A-D) *Pegl-9::GFP* expression in adult animals under normal culture conditions and heat shock. Animals are shown as DIC images (A and C) and corresponding images of GFP fluorescence (B and D). (A and B) Under normal conditions, *Pegl-9::GFP* was expressed in the body muscle, vulva, pharynx, anterior intestine, rectal cells and additional cells in the tail. (C and D) After heat shock treatment (29°C for 20 hours), *Pegl-9::GFP* was strongly induced in the intestine.
