# Supplementary material for: Crosstalk in oxygen homeostasis networks: SKN-1/NRF inhibits the HIF-1 hypoxia-inducible factor in Caenorhabditis elegans
Source: PLoS One. 2021 Jul 9;16(7):e0249103. doi: 10.1371/journal.pone.0249103 (PMC8270126; doi:10.1371/journal.pone.0249103)

S3 Fig. Heat shock induced another independent *Pegl-9::GFP* transgenic line in animals carrying the wild-type *skn-1* allele, but did not induce the reporter in animals carrying the *skn-1(zu67)* loss-of-function mutation.

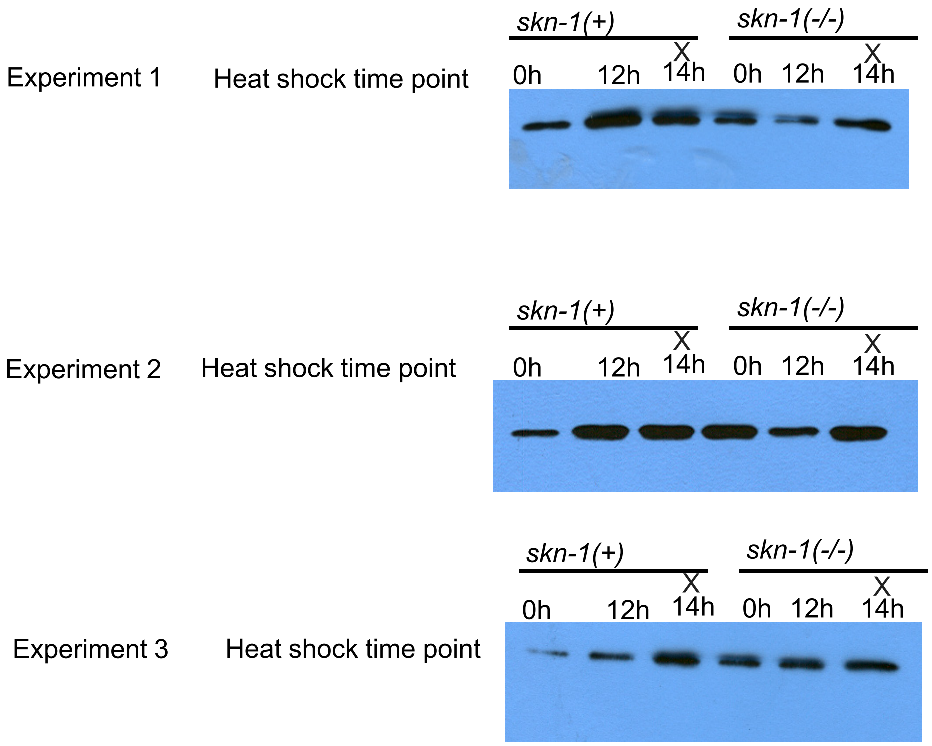

Supplement: S3 Fig — (PDF) [file pone.0249103.s003.pdf]
