# Supplementary material for: Crosstalk in oxygen homeostasis networks: SKN-1/NRF inhibits the HIF-1 hypoxia-inducible factor in Caenorhabditis elegans
Source: PLoS One. 2021 Jul 9;16(7):e0249103. doi: 10.1371/journal.pone.0249103 (PMC8270126; doi:10.1371/journal.pone.0249103)

S4 Fig. Heat shock increased the expression of *Pegl-9*::*GFP*, but did not increase the expression of the reporter in which the putative SKN-1 binding site was mutated (*P(m)egl-9::GFP*) in another independent line.

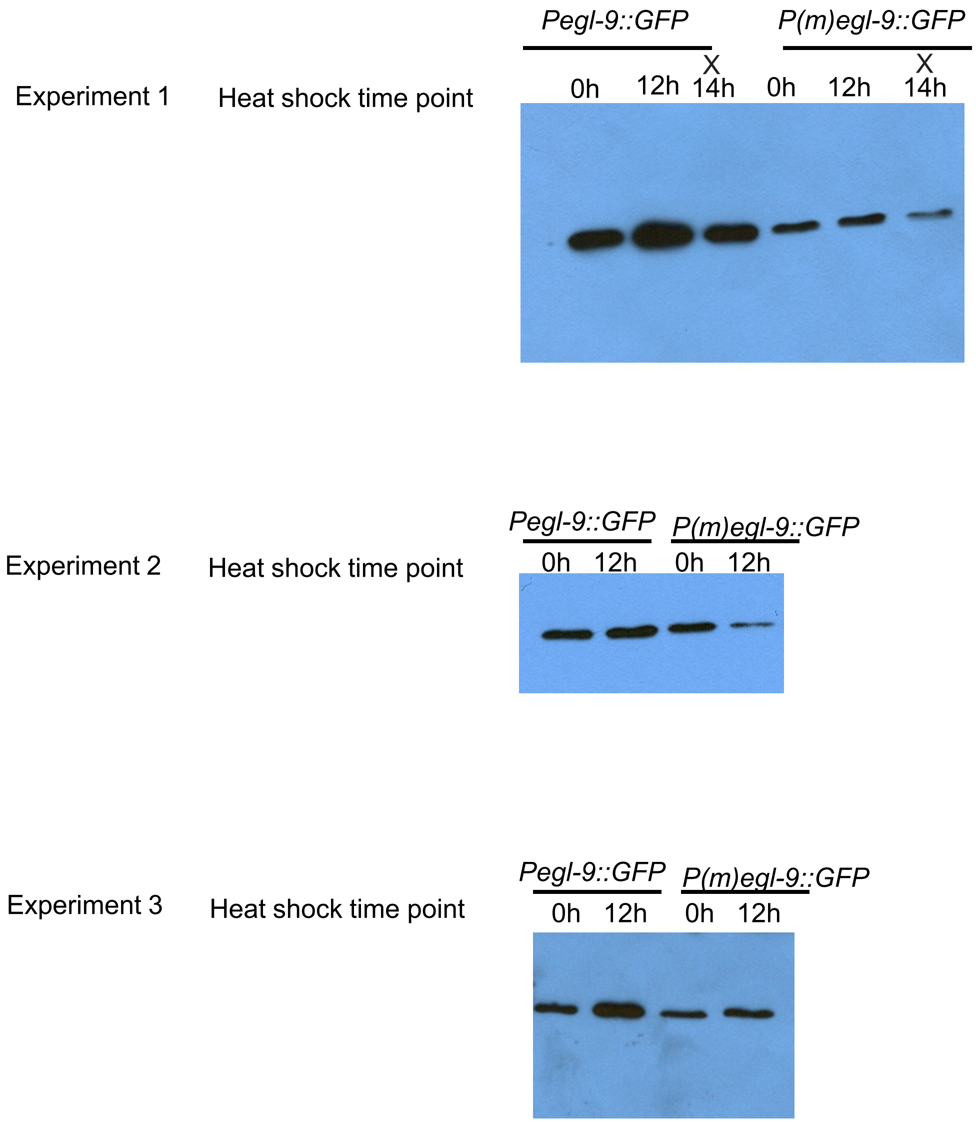

Supplement: S4 Fig — (PDF) [file pone.0249103.s004.pdf]
