# Supplementary material for: Crosstalk in oxygen homeostasis networks: SKN-1/NRF inhibits the HIF-1 hypoxia-inducible factor in Caenorhabditis elegans
Source: PLoS One. 2021 Jul 9;16(7):e0249103. doi: 10.1371/journal.pone.0249103 (PMC8270126; doi:10.1371/journal.pone.0249103)
Supplement: S5 Fig — (A) RNAi for sams-1 (S-adenosyl methionine synthetase) increased Pnhr-57::GFP expression more than 7-fold in animals carrying the wild-type hif-1 allele relative to control RNAi, and increased the reporter 3-fold in animals carrying the hif-1(ia04) deletion. The difference in RNAi effect between hif-1(+) and hif-1(ia04) strains is statistically significant (*p < 0.05, from six independent experiments, by student t-test). (B) RNAi for the SREBP homolog sbp-1 increased expression of the reporter more than 3-fold in animals carrying the wild-type hif-1 allele, but had no effect on Pnhr-57::GFP expression in hif-1(ia04) mutants. The difference in RNAi effect between hif-1(+) and hif-1(ia04) strains is statistically significant (*p < 0.05, from five independent experiments, by student t-test). GFP levels were determined by protein blots, and the control animals were fed on bacteria carrying the empty RNAi vector (L4440). The experiments were conducted in RNAi-sensitive strains (rrf-3(pk1426)). (DOCX) [file pone.0249103.s005.docx]

**S5 Fig. RNAi inactivation of *sams-1* or*sbp-1* increased *Pnhr-57::GFP*expression.**

(A) RNAi for *sams-1* (S-adenosyl methionine synthetase) increased *Pnhr‑57:*:*GFP* expression more than 7-fold in animals carrying the wild-type *hif-1* allele relative to control RNAi, and increased the reporter 3-fold in animals carrying the *hif-1(ia04)* deletion. The difference in RNAi effect between *hif-1(+)* and *hif-1(ia04)* strains is statistically significant (**p* < 0.05, from six independent experiments, by student *t-*test). (B) RNAi for the SREBP homolog *sbp-1* increased expression of the reporter more than 3-fold in animals carrying the wild-type *hif-1* allele, but had no effect on *Pnhr-57::GFP* expression in *hif-1(ia04)* mutants. The difference in RNAi effect between *hif-1(+)* and *hif-1(ia04)* strains is statistically significant (**p* < 0.05, from five independent experiments, by student *t-*test). GFP levels were determined by protein blots, and the control animals were fed on bacteria carrying the empty RNAi vector (L4440). The experiments were conducted in RNAi-sensitive strains (*rrf-3(pk1426*)).
