# Supplementary material for: Crosstalk in oxygen homeostasis networks: SKN-1/NRF inhibits the HIF-1 hypoxia-inducible factor in Caenorhabditis elegans
Source: PLoS One. 2021 Jul 9;16(7):e0249103. doi: 10.1371/journal.pone.0249103 (PMC8270126; doi:10.1371/journal.pone.0249103)
Supplement: S2 Table — (DOCX) [file pone.0249103.s007.docx]

**S2 Table. Genes for which RNAi caused *hif-1*-dependent increase of *Pnhr-57::GFP* expression.**

|  | **Gene** | **Description** | ***Pnhr-57::GFP* expression pattern caused by RNAi** | |
| --- | --- | --- | --- | --- |
|  |  |  | ***hif-1 (+)*** | ***hif-1 (ia04)*** |
| Mitochondria  l/ Metabolism | T09B4.9 | Mitochondrial import inner  membrane translocase | Strong, in the gut | Weak, in the gut |
|  | *sdhb-1* | Succinate dehydrogenase subunit | Weak, in the gut | Dim, in the gut |
|  | *sams-1* | S-adenosyl methionine synthetase | Strong, in the gut; and weak, in the hypodermis | Strong, in the gut |
|  | *sco-1* | Putative cytochrome C oxidase  assembly protein | Weak, in the gut | Dim, in the gut |
|  | W02F12.5 | Oxoglutarate dehydrogenase complex | Strong, in the gut | Weak, in the gut |
| Protein  turnover | *rpn-11* | Proteasome regulatory particle | Strong, in the gut | Weak, in the gut |
|  | *rpn-12* | Proteasome regulatory particle | Strong, in the gut | Only in the posterior gut |
| Transcription/  translation | *rrt-2* | Arginyl-tRNA synthetase | Strong, in the gut | Weak, in the gut |
|  | *sbp-1* | Transcription factor: SREBP  homolog | Strong, in the gut | Dim, in the gut |
|  | *skn-1* | Transcription factor: Nrf homolog | Weak, in the gut | Dim, in the gut |
| Known  regulators of  HIF-1 | *vhl-1* | E3 ligase, Von Hipple Lindau | Strong, in the gut | Dim, in the gut |
|  | *egl-9* | Prolyl hydroxylase | Strong, in the gut* | Dim, in the gut |
|  | *rhy-1* | Acyltransferase | Strong, in the gut and  hypodermis | Dim, in the gut |

*Strong loss-of-function mutations in *egl-9* cause *Pnhr-57*::*GFP* expression in the gut and hypodermis. This *egl-9* RNAi clone caused a partial loss-of-function phenotype.
