# Supplementary material for: Crosstalk in oxygen homeostasis networks: SKN-1/NRF inhibits the HIF-1 hypoxia-inducible factor in Caenorhabditis elegans
Source: PLoS One. 2021 Jul 9;16(7):e0249103. doi: 10.1371/journal.pone.0249103 (PMC8270126; doi:10.1371/journal.pone.0249103)

Raw western blot data for Fig 2A: *skn-1* RNAi increased expression of the *Pnhr-57::GFP* reporter.

Experiment 1

Hypoxia time point

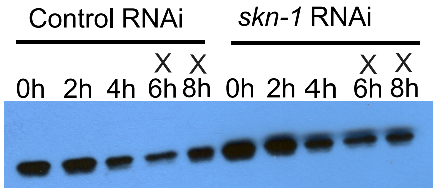

Experiment 2

Hypoxia time point

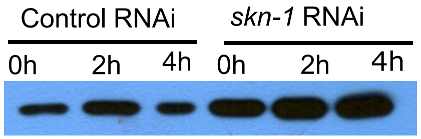

Experiment 3: representative gel for Fig 2A

Hypoxia time point

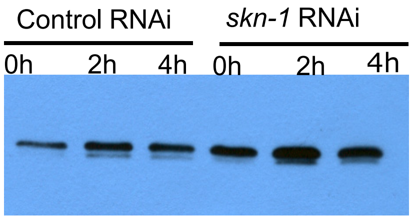

Raw western blot data for Fig 2B: *skn-1* RNAi increased HIF-1 protein levels.

Control RNAi | *skn-1(1)* RNAi | *skn-1(2)* RNAi

Experiment 1: representative gel for Fig 2B

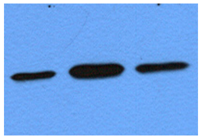

Control RNAi | *skn-1(1)* RNAi | *skn-1(2)* RNAi

Experiment 2

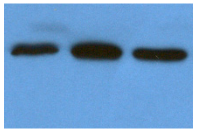

Control RNAi | *skn-1(1)* RNAi | *skn-1(2)* RNAi

Experiment 3

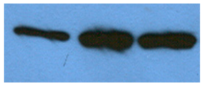

Raw western blot data for Fig 5A: Heat shock induced *Pegl-9::GFP* in animals carrying the wild-type *skn-1* allele, but did not induce the reporter in animals carrying the *skn-1(zu67)* loss-of-function mutation.

Experiment 1 Heat shock time point

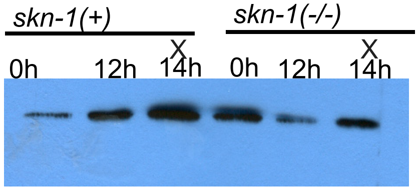

Experiment 2 Heat shock time point

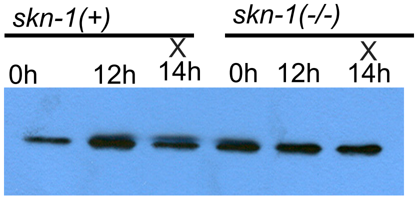

Experiment 3: Representative gel for Fig 5A Heat shock time point

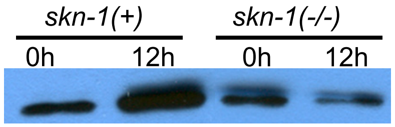

Experiment 4 Heat shock time point

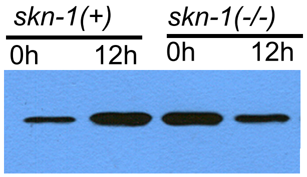

Raw western blot data for Fig 5B: Heat shock increased the expression of *Pegl-9::GFP*, but did not increase the expression of the reporter in which the putative SKN-1 binding site was mutated (*P(m)egl-9::GFP*).

Pegl-9::GFP | *P(m)egl-9::GFP*

X X | X X

0h 12h 14h 16h | 0h 12h 14h 16h

Experiment 1 — Heat shock time point

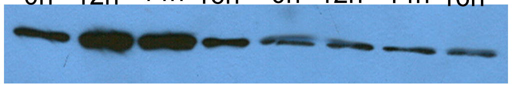

Pegl-9::GFP | *P(m)egl-9::GFP*

X X | X X

0h 12h 14h 16h | 0h 12h 14h 16h

Experiment 2 — Heat shock time point

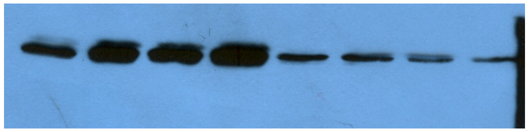

Pegl-9::GFP | *P(m)egl-9::GFP*

X X | X X

0h 12h 14h 16h | 0h 12h 14h 16h

Experiment 3 — Heat shock time point

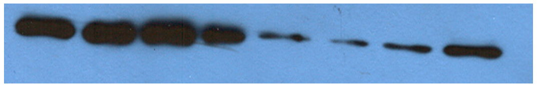

Pegl-9::GFP | *P(m)egl-9::GFP*

X | X

0h 12h 14h | 0h 12h 14h

Experiment 4 — Heat shock time point

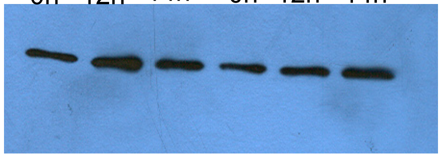

Pegl-9::GFP | *P(m)egl-9::GFP*

0h 12h | 0h 12h

Experiment 5: representative gel for Fig 5B — Heat shock time point

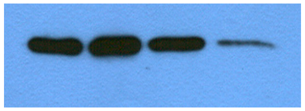

Raw western blot data for Fig 6: *gsk-3 RNAi induction of Pegl-9::GFP* and P*(m)egl-9::GFP*

Experiment 1

Pegl-9::GFP

Control RNAi | X 88h | X 96h | 102h | X 120h

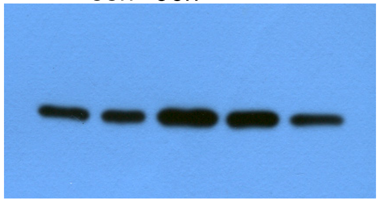

P(m)egl-9::GFP

Control RNAi | X 88h | X 96h | 102h | X 120h

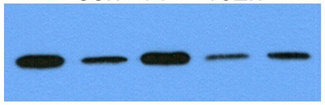

Experiment 2

Pegl-9::GFP — Control RNAi | 102h | X 120h

P(m)egl-9::GFP — Control RNAi | 102h | X 120h

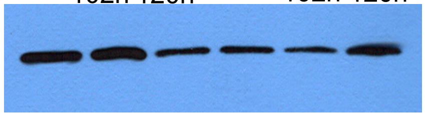

L4 worms were put on *gsk-3* RNAi plates or control RNAi plates to lay eggs at o hour. Sample L4 worms for western blot at different time points.

Experiment 3

Pegl-9::GFP — Control RNAi | 102h

P(m)egl-9::GFP — Control RNAi | 102h

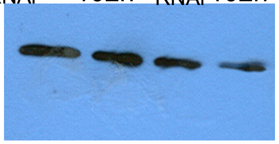

Experiment 4: representative gel for Fig 6

Pegl-9::GFP — Control RNAi | 102h

P(m)egl-9::GFP — Control RNAi | 102h

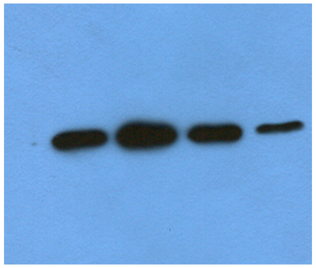

Supplement: S1 Raw images — (PDF) [file pone.0249103.s008.pdf]
